# Unbiased assessment of APR-246 responsive p53 mutants in ovarian cancer

**DOI:** 10.1038/s41420-026-03152-5

**Published:** 2026-05-19

**Authors:** Anais Saunders, Caili Tong, Anthony N. Karnezis, Gary S. Leiserowitz, Jeremy Chien

**Affiliations:** 1 Department of Biochemistry and Molecular Medicine, University of California Davis, Sacramento, CA USA; 2 Department of Pathology and Laboratory Medicine, University of California Davis, Sacramento, CA USA; 3 Department of Obstetrics and Gynecology, University of California Davis, Sacramento, CA USA

**Keywords:** Ovarian cancer, Targeted therapies, Cancer genetics

## Abstract

*TP53* is the most frequently mutated gene in high-grade serous ovarian cancer, and the majority of these mutations result in overexpression of mutant p53. Therefore, therapies that can restore the wild-type activities from p53 mutants have the potential to cause p53-mediated tumor suppressive effects. Accordingly, prior studies have shown that APR-246 can bind to specific p53 mutants, restore wild-type activities, and cause tumor suppression in cancer cell lines with hot-spot mutations. However, systematic analysis of what other p53 mutants are responsive to APR-246 is currently lacking. Here, we used a *TP53* mutagenesis library to perform functional genetic screens to identify *TP53* mutants that are sensitive to APR-246. Our studies confirm weak p53-dependent cytotoxic/cytostatic effects in OVCAR5 but not in SKOV3 and CAOV3. Further analysis identified ferroptosis sensitivity as a major determinant of APR-246 sensitivity. This agrees with the known effect of APR-246 as a disruptor of the redox system. In ferroptosis-sensitive SKOV3 and CAOV3, p53-dependent effects are not observable because APR-246 acts primarily through its induction of ferroptosis. Only in OVCAR5, which is relatively insensitive to ferroptosis compared to SKOV3 and CAOV3, do we observe a p53-dependent effect of APR-246. This study demonstrates that p53-specific effects of APR-246 are only observable in a narrow dosing window, under a specific set of conditions. Collectively, our results highlight the limitation of APR-246 as a p53-rescuing drug because of its overwhelming off-target effects on the redox system at doses that are sufficient to induce ferroptosis but insufficient to rescue mutant p53.

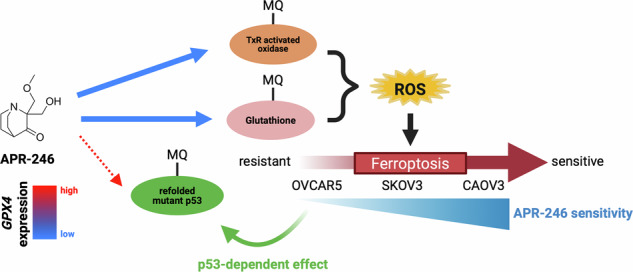

## Introduction

*TP53* is the most frequently mutated tumor suppressor gene across human cancers [1, 2]. Mutation rate varies among cancer subtypes, with some, such as high-grade serous ovarian cancer (HGSOC), reaching upwards of 96% [3]. HGSOC remains the most deadly gynecological cancer in the US and often faces difficulty in treatment success due to its late-stage diagnosis [4]. Despite being a tumor suppressor, mutated *TP53* is notorious for its gain-of-function (GOF) activity, resulting from changes in transcriptional activity of the protein that render it pro-oncogenic. This, combined with the widespread prevalence of mutated p53 in human cancers, makes it an ideal candidate for targeted therapy. Unlike most targeted therapies, which act by inhibiting their target, a p53-targeted therapy must reactivate the protein, restoring its wild-type (WT)-like activity. It is well appreciated that certain mutant p53 proteins exhibit stark decreases in the stability of their core DBD domain [5–7]. Only three compounds that belong to the p53-reactivation or p53-stabilization class of targeted therapies have made it to clinical trials [8].

Eprenetapopt/APR-246/PRIMA-1^MET^ is a small molecule drug which has been proposed to bind to cystine residues in mutant p53, thermodynamically stabilizing it such that both WT conformation and functionality is restored [9]. Despite the numerous possible p53 mutations that exist, most studies with APR-246 have focused on the “hotspot” mutations, namely the conformational mutant R175H [10–13]. While “hotspot” mutants account for nearly 30% of all p53-mutations, 70% of mutants remain unexplored [14]. To our knowledge, there has yet to be systematic functional genomic screening for the identification of APR-246 rescuable p53 mutants. Furthermore, there have not been any studies that characterize the effect of P72R, the most common germline single-nucleotide polymorphism (SNP) in the *TP53* gene, on mutant p53’s response to APR-246.

Our study addresses these gaps in clinically actionable knowledge in two ways. To identify p53 mutants that are amenable to rescue by APR-246, we conducted a functional genetic screen of >8000 p53 variants against the p53-rescue drug. Such a wide-scale approach has not yet been conducted. Additionally, we assessed the role of the P72R SNP in impacting the rescue potential of p53 variants, which has not been addressed thus far in the literature. The P72R SNP has previously been described as differentially impacting somatically mutated p53 proteins on a functional and potentially structural level [15–18]. Altogether, this study assesses the activity of APR-246 as a pan-mutant p53 reactivator and evaluates the role of the P72R SNP on rescue by APR-246.

## Results

### Functional genetic screen of TP53 mutants against APR-246

**Fig. 1:**
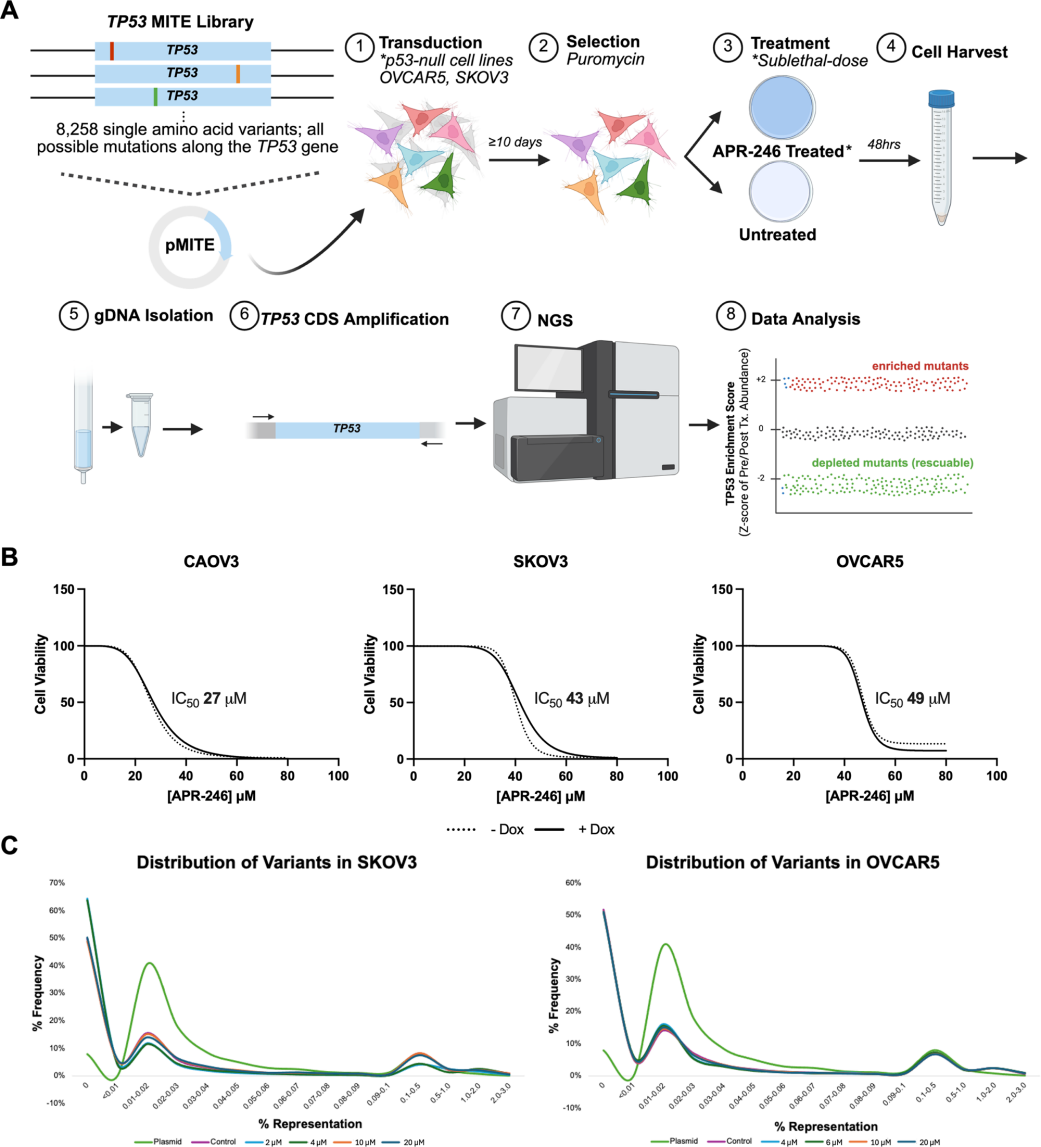
Functional genetic screen of *TP53* mutants against APR-246. **A** Representative graphic of an experimental approach to a functional genetic screen. **B** Dose-response of p53-null parental ovarian cancer cell lines. Cells were treated for 72 hours prior to the Cell Titer Glo cell viability assay. Data are shown as % cell viability relative to the vehicle and fit to a sigmoidal 4PL model. Mean IC_50_ (*n* = 3). **C** Summary statistics for each experimental group in the functional screen, where % representation indicates variant representation within the population and % frequency indicates the % of variants that lie within that representation range.

To identify APR-246-sensitive *TP53* mutants, we performed functional genetic screenings using a *TP53* mutagenesis library, MITE. This pooled lentiviral library was generated by the Hahn lab and contains 8258 amino acid variants, spanning the entirety of the *TP53* gene [19]. The MITE lentiviral library was stably transduced into p53-null ovarian cancer cell lines, SKOV3 and OVCAR5, and transduced cells were selected for using puromycin (Fig. 1A). MITE-expressing cell lines were then split into two populations, one of which remained untreated, and the other which was treated with a pre-determined sublethal dose of APR-246 (Fig. 1A). Following a 48 hr treatment period, cells were harvested, and the genomic DNA was isolated from each sample (Fig. 1A). The TP53 coding sequence was then amplified, and the amplicons underwent next-generation sequencing (Fig. 1A). We predicted that upon rescue by APR-246, sensitive *TP53* mutants would regain apoptotic activity and self-select themselves out of the variant population. Analysis of the variant representation in treated vs untreated samples would allow us to determine variants that were depleted in the treated population as compared to the control (Fig. 1A).

Given that APR-246 is a small-molecule drug, it is expected to have off-target, non-p53-dependent effects. These have been previously described as inhibition of the antioxidant defense system, leading to an increase in oxidative stress [20]. In order to mitigate these off-target effects, we sought to perform our pooled functional depletion screenings against sublethal doses of APR-246. These thresholds were determined in p53-null parental cell lines CAOV3, SKOV3, and OVCAR5, and IC_50_ values were 27, 43, and 49 μM, respectively (Fig. 1B). Due to the intrinsic sensitivity of CAOV3 to APR-246, we chose to perform our screenings in SKOV3 and OVCAR5 at doses ranging from 2-20 μM.

Summary statistics show a similar distribution of variants in both the plasmid input and the stably transduced cell lines (Fig. 1C). It should be noted that upon transduction of the cells with the variant library, there was a ~50% loss in total variant representation (Fig. 1C; Supplementary Fig. 1A). Due to the constitutive expression of this variant library, we predict that this population is likely representative of silent variants that exhibit WT-like p53 activity and are therefore dropped out of the population. We also noted two distinct populations of mutants with varying representation. The major population has an average representation between 0.01-0.02%, and the minor population has a higher representation level between 0.5–1% (Fig. 1C). We questioned the identity of variants within the minor population, hypothesizing that it may be made up of known gain-of-function or hot-spot p53 mutants. However, the representation of hot-spot mutants within the variant library was not particularly enriched in either the SKOV3 or OVCAR5 cell lines (Supplementary Fig. 1B). We also noted that these major and minor populations were observed in the plasmid input (Fig. 1C), suggesting that this was not a result of selection in transduced cells but perhaps a result of selection during amplification of the original plasmid library in *E. Coli*.

Likewise, variant distribution in APR-246-treated samples largely resembled the distribution in control populations. We ensured only viable cells were harvested following treatment to reduce the chance of sequencing false-positive variants (Supplementary Fig. 1C). We anticipated the potentially sensitive population of variants to represent a small fraction of the total MITE variant population, resulting in little to no observable shift in the overall variant representation distribution (Fig. 1C). Despite this, we did observe a dose-dependent response to APR-246 following treatment of the MITE transduced cells (Supplementary Fig. 1C).

### Selection of candidate mutants

**Fig. 2:**
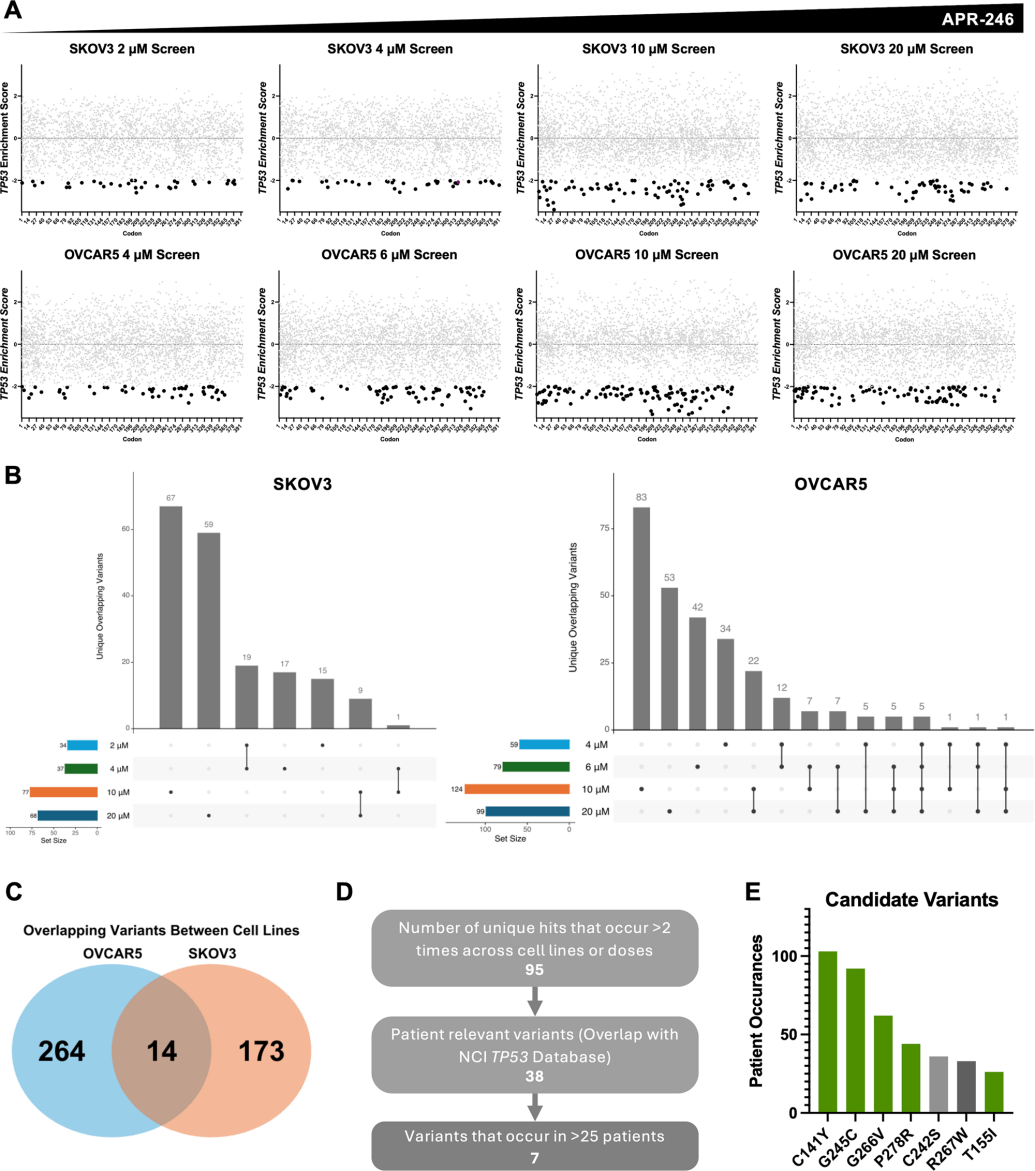
Selection of candidate mutants. **A** Changes in variant representation following treatment with increasing doses of APR-246 in functional screen. TP53 Enrichment Score represents Z-score-normalized values for each variant, calculated relative to the mean and standard deviation of all variants at that codon. Variants with an enrichment score <-2 are bold and indicate the population of interest for follow up. **B** Overlap analysis between variants with an enrichment score of <-2 from the various treatment groups for each respective cell line. **C** Overlap analysis between variants with an enrichment score of <-2 from each cell line. **D** Workflow for the identification of the most clinically relevant variants with an enrichment score of <-2. **E** Number of occurrences of each of the 7 candidate variants that occur in >25 patients, according to the NCI TP53 database. Those in green were chosen for functional follow-up due to higher prevalence with the exception of T155I, which was selected due to its overlap across >2 groups.

Upon massively parallel sequencing of our samples, we determined the enrichment and depletion of each variant within the treatment populations relative to the untreated control population. Each variant received a TP53 enrichment score wherein a negative *TP53* enrichment score is representative of a variant which was depleted in the treatment population as compared to the untreated control population (Fig. 2A). A threshold *TP53* enrichment score of -2 or less was used to identify variants which were significantly depleted in treated populations (Fig. 2A). There was an observable increase in the number of variants which fell below this threshold upon increase in APR-246 concentration, in both SKOV3 and OVCAR5 (Fig. 2A). We noted that the prototypical conformational mutant R175H, which has previously been characterized as responsive to APR-246 [10], was neither significantly enriched nor depleted in the treated population (Supplementary Fig. 1E). We hypothesized that due to the use of sublethal doses in our screen we may be selecting variants which are ultra-sensitive to APR-246.

Hit prioritization was carried out by performing overlap analysis between significantly depleted variants from each treatment population (Fig. 2B). Overall, SKOV3 had a smaller population of 187 significantly depleted variants, while OVCAR5 had 278. Likewise, much less overlap was observed in the SKOV3 cell line than was observed in the OVCAR5 cell line (Fig. 2B). We also looked at the overlap between our two cell lines with a small population of 14 variants which overlapped (Fig. 2C). In total, 95 variants were identified as occurring at least twice across two treatment groups or two cell lines (Fig. 2D). Further stratification of candidate variants was performed via a final overlap analysis between our 95 unique variants and the NIH *TP53* Database. This revealed 38 variants that were both identified in our screen and characterized in the database as non-functional, damaging mutations (Fig. 2D). Further, we determined that 7 of these variants occurred in more than 25 patients (Fig. 2D, E). These represent the most clinically relevant hits in our screen. We chose to perform functional studies on five of these variants, C141Y, T155I, G245C, G266V, and P278R (Fig. 2E).

### Validation of candidate mutants

In addition to somatic mutations in the *TP53* gene, germline single-nucleotide polymorphisms (SNPs) are also commonly found in the population. The most common intragenic coding SNP is the Proline to Arginine polymorphism at residue 72 (P72R), within the proline-rich domain of the gene [18, 21]. In WT p53, the P72R SNP has not been observed to cause structural differences in the protein [22]. However, many studies have demonstrated that even in WT p53, the P72R SNP exerts an effect on its transcriptional regulation of target genes and subsequent activation of biological programs [16, 17, 22–25]. In the presence of secondary somatic mutations, the P72R SNP has been shown to exert differential effects on tumorigenesis that is dependent on SNP identity [25]. Prior studies in the Chien lab, consisting of DNA sequence analysis of over 4000 cancer samples, have shown a selection bias against missense mutants carrying the P72 SNP, and cancer patients harboring somatic *TP53* mutations in the P72 allele showed improved prognosis [15]. This effect has also been demonstrated in both in vitro cell culture models and in vivo mouse models. Additionally, studies from the Chien lab have shown that somatic mutants with P72 SNP are more immunoreactive to antibodies against WT p53, suggesting the higher propensity of these mutants to assume WT conformation as compared to their R72 counterparts [15]. Structural modeling studies further support the idea that the P72R SNP may affect the N-terminal domain flexibility and consequently functionality of the p53 protein [15]. Due to the conformational differences between the P72 and R72 missense mutants, we speculated that their propensity for rescue by p53 targeting drugs, such as APR-246, may differ in that mutants with the P72 SNP would exhibit increased sensitivity.

**Fig. 3:**
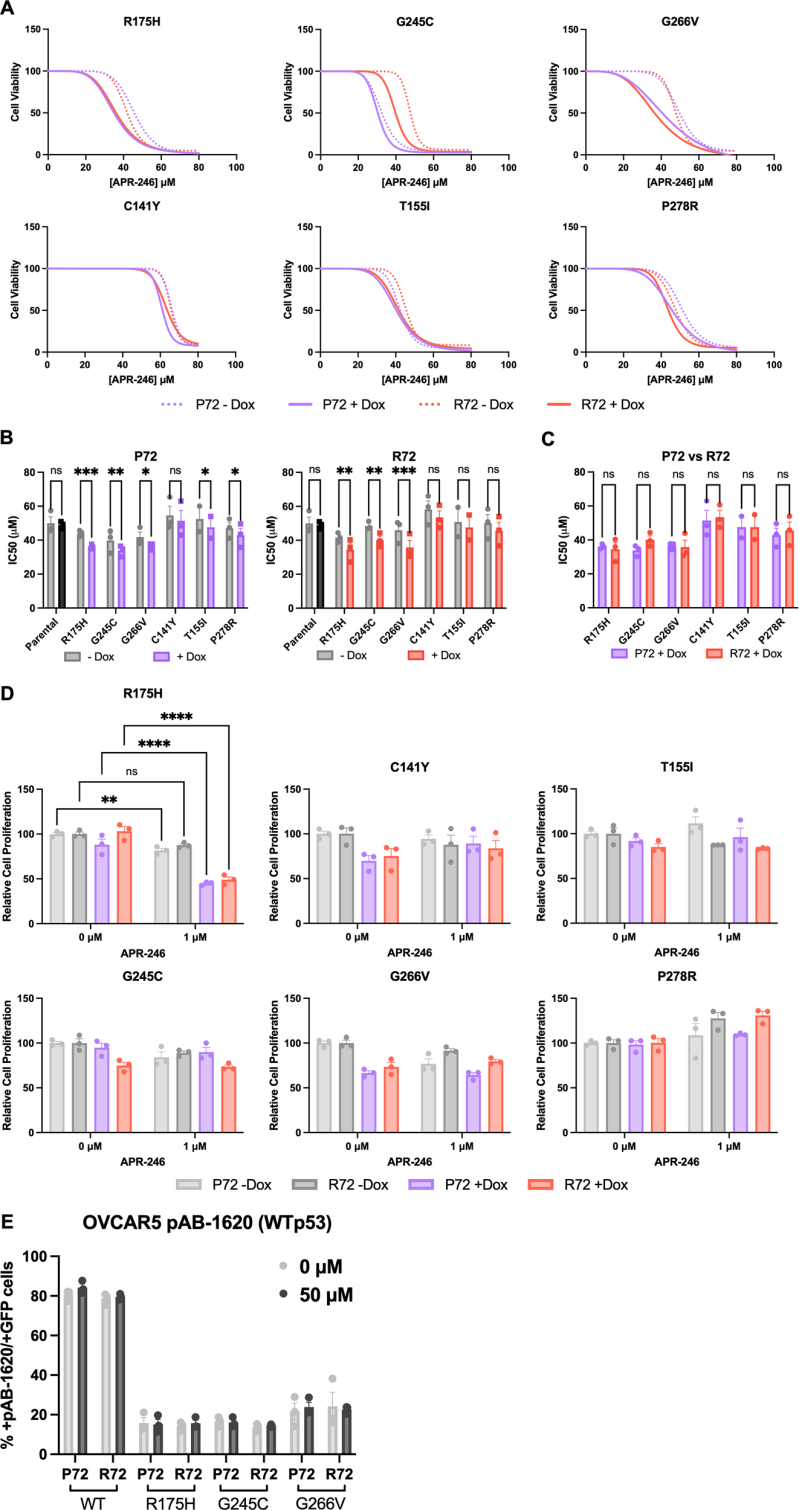
Validation of candidate mutants. **A** Dose-response of candidate expressing-OVCAR5 cells generated under Tet-off (dotted line) and Tet-on (solid line) conditions. Cells were treated for 72 hours prior to the Cell Titer Glo cell viability assay. Data are shown as % cell viability relative to the vehicle and fit to a sigmoidal 4PL model. **B** IC_50_ values for each candidate variant under Tet-off (grey) or Tet-on (color) conditions. Mean IC_50_ + SEM (*n* = 3*)*. **C**) IC_50_ values for each candidate variant under Tet-off (grey) or Tet-on (color) conditions. Mean IC_50_ + SEM (*n* = 3*). Statistical analysis was performed using a 2-way ANOVA with multiple comparisons between P72 and R72 groups. **D** Colony formation assay of candidate expressing-OVCAR5 cells under Tet-off (grey) and Tet-on (color) conditions. Cells were treated for 14-days prior SRB staining. Data are shown as % cell proliferation relative to the Tet-off group (*n* = 3).* **E** Immunofluorescence of variant-expressing-OVCAR5 cells with WT-recognizing pAb-1620. Data is shown as the % of pAb-1620 positive cells among the GFP-positive (p53-expressing) cell population. Cells were treated with 50 μM APR-246 for 6 h prior to fixation and staining (*n* = 3). **Statistical analysis was performed using a 2-way ANOVA with multiple comparisons between Tet-on and Tet-off groups. ns = non-significant, *p < 0.05, **p < 0.005, ***p < 0.0005. *(T155I *n* = 2).

To validate our candidate variants and test the effect of the P72R SNP, we cloned each of our 5 variants, C141Y, T155I, G245C, G266V, and P278R, into doxycycline-inducible vectors containing either the P72 or R72 SNP. We included R175H, a hotspot mutant that has been previously published as sensitive, as our positive control. Each of these paired variants was transduced into OVCAR5 cells, selected for, and expanded. We began our validation by performing 72 hr dose-response assays against APR-246 in the presence or absence of doxycycline (Fig. 3A). We observed a consistent increase in sensitivity, or reduced IC_50_ value, for mutant cells treated with doxycycline but not parental OVCAR5 cells, indicating that induction of mutant p53 sensitized cells to APR-246 (Fig. 3A, B). Additionally, three of the six variants exhibited greater sensitivity than the parental p53-null OVCAR5 cells, including the positive control R175H (Fig. 3B; Supplementary Fig. 2A). Comparison of variant sensitivity to APR-246 in the presence of the P72 or R72 SNP revealed no significant difference between the SNPs (Fig. 3C). We noted that the effect size between non-induced and induced cells was relatively weak (Fig. 3A, B). We assessed the expression levels of the p53 variants in our transduced cell lines, using flow cytometry, and found that in the uninduced state ~5–8% of cells had some p53 expression while in the induced state ~40–55% of cells lacked p53 expression (Supplementary Fig. 2B). We predict that the presence of non-transduced cells in the induced population could contribute to an attenuation of the mutant p53 response to APR-246. While these short-term dose-response assays assess the cytotoxic effect of APR-246, we were also interested in the potential cytostatic effects of APR-246. To assess this, we performed long-term, 14-day, clonogenic assays. We observed that induction of R175H expression in OVCAR5 cells resulted in a more significant reduction in cell proliferation upon treatment with APR-246 (Fig. 3D, Supplementary Fig. 2C, D). However, this effect was not seen in any of the other candidate variants (Fig. 3D). Additionally, we performed immunofluorescence studies, following treatment with a 50 μM dose of APR-246, using the WT-recognizing antibody pAB-1620. WT-expressing cells were included as a positive control for staining, with an average of ~80% of GFP-positive/p53-positive cells staining positive for pAB-1620 (Fig. 3E, Supplementary Fig. 2D, E). Mutant p53-expressing cells exhibited significantly lower levels of staining by pAB-1620, indicative of a loss of WT conformation (Fig. 3E, Supplementary Fig. 2D, E). More importantly, a 24-hr 50 μM APR-246 treatment had no effect on recognition of mutant p53 by pAB-1620 (Fig. 3E, Supplementary Fig. 2D, E). This is in contrast to previous studies [10, 26].

### Validation of conformational mutant R175H in ovarian cancer cell lines

**Fig. 4:**
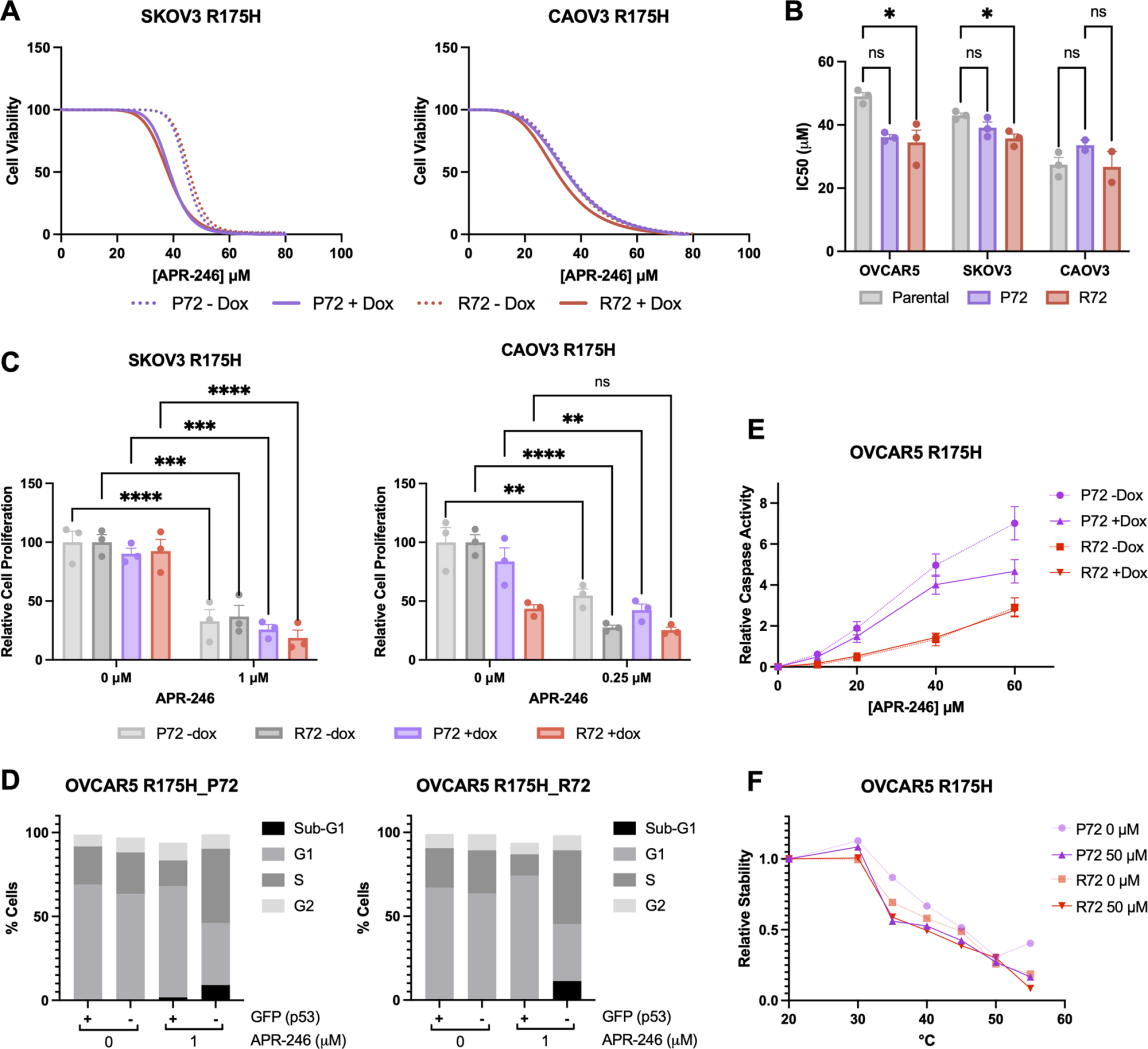
Validation of conformational mutant R175H in ovarian cancer cell lines. **A** Dose-response of R175H expressing cells generated under Tet-off (dotted line) and Tet-on (solid line) conditions. Cells were treated for 72 hours prior to the Cell Titer Glo cell viability assay. Data are shown as % cell viability relative to the vehicle and fit to a sigmoidal 4PL model. **B** IC_50_ values for R175H-expressing cell lines. Mean IC_50_ + SEM (*n* = 3*). Statistical analysis was performed using a 2-way ANOVA with multiple comparisons to respective parental groups. **C** Colony formation assay of R175H expressing cells under Tet-off (grey) and Tet-on (color) conditions. Cells were treated for 14-days prior SRB staining. Data are shown as % cell proliferation relative to the Tet-off, vehicle-treated group (*n* = 3). Statistical analysis was performed using a 2-way ANOVA with multiple comparisons to the vehicle-treated group. **D** Cell cycle analysis via flow cytometry. Cells were treated for 48 hours prior to processing. **E** Cells were treated with APR-246 for 72 hours under Tet-on (dotted) and Tet-off (solid) conditions before undergoing caspase Glo assay. **F** Cellular thermal shift assay following 4 h data are shown as protein stability relative to non-heated control. *p < 0.05, **p < 0.005, ***p < 0.0005, ****p < 0.0001. *(CAOV3 R175H_P72/R72 *n* = 2).

APR-246 was previously reported to cause p53-independent effects through ferroptosis by affecting the redox capacity of cancer cells [27–29]. To discern p53-dependent and independent effects of APR-246 and a potential role of germline SNP at codon 72 in modifying these effects, we utilized three p53-null ovarian cancer cell lines expressing inducible R175H mutant with either P72 or R72 SNP. We chose three cell lines, OVCAR5, SKOV3, and CAOV3, because they exhibit different intrinsic sensitivity to APR-246 (Fig. 1B). Interestingly, the introduction of the R175H mutation in these cell lines yields variable results. Short-term dose response assays show that OVCAR5 cells expressing R175H are slightly more sensitive to APR-246 compared to parental cells (Fig. 4A, B), indicated by a ~30% reduction in the IC_50_ upon expression of R175H. We observe a ~15% reduction in IC_50_ in SKOV3 and no reduction in CAOV3 cells when R175H transduced cells were compared with parental cells (Fig. 4B). We also performed long-term growth assays in R175H expressing SKOV3 and CAOV3 and found that cell proliferation was significantly reduced in both the uninduced and induced state (Fig. 4C). These results indicate that APR-246 induces p53-independent effects in these cells and that these off-target effects prevent us from detecting any mutant-specific effects even at low doses. We focused our efforts on identifying the mechanism by which APR-246 induces a p53-specific effect in OVCAR5 expressing R175H (Fig. 4D).

Due to the nature of a clonogenic assay, a measure of cellular proliferation, we investigated the effect of a 1 μM APR-246 treatment on cell cycle dynamics. Treatment with APR-246 affected p53-expressing and p53-null cell lines differently such that p53-expressing cells underwent little to no changes in cell cycle distribution (Fig. 4D). On the other hand, p53-null OVCAR5 cells exhibited a decrease in G1 and an increase in S phase (Fig. 4D). Additionally, a Sub-G1 population of cells was present, likely indicative of cells undergoing cell death (Fig. 4D). The P72R SNP had no effect on cell cycle dynamics (Fig. 4D). Neither APR-246 treatment nor the P72R SNP had any effect on R275H expressing SKOV3 cells (Supplementary Fig. 3A). An additional study with 100 and 200 μM doses of APR-246 showed a dose-dependent decrease in G1 that is both p53 and P72R-independent (Supplementary Fig. 3B). Further, APR-246 treatment at this higher dose was found to have no differential effect on cell doubling times of p53-expressing vs p53-null cells (Supplementary Fig. 3C).

While functional rescue of the R175H mutant was observable via a selective increase in G1 arrest in p53-expressing OVCAR5 cells (Fig. 4D), we did not observe an increase in p21 expression upon treatment with MQ, the active metabolite of APR-246 (Supplementary Fig. 3D). We postulate that this is due to the mixed population of p53-expressing and p53-null cells in the induced state. While our cell cycle analysis allows us to discriminate between these populations on a single cell level, the western blot does not, potentially muting the effects observed in the p53-expressing cells. While APR-246 treatment did cause dose-dependent increase in caspase activity, this appeared to be independent of p53-expression (Fig. 4E). The P72R SNP did seem to exert a differential effect on caspase induction, with the R72 SNP exhibiting lower levels than the P72 SNP (Fig. 3E). This agrees with previous observations that indicate p53 mutants with the P72 SNP have a greater propensity to retain WT-like p53 activity in comparison to those with the R72 SNP [15, 25, 30–32].

Upon further investigation using flow cytometry and the p53 WT-recognizing pAb-1620, we noted that APR-246 treatment caused a 50% decrease in the pAb-1620-positive cell population (Supplementary Fig. 3E). It is important to note that these cells only account for about 10% of the total population, which explains why these mutant-specific effects are difficult to discern when observing effects on the cell population as a whole. We also noted that about 20% of the R175H mutants were recognized by the pAb-1620. We hypothesize that this is due to our use of the R175H mutant in cooccurrence with the P72 SNP, which we have previously demonstrated has a propensity to be recognized by this antibody [33].

The stability of the R175H p53 protein upon treatment with APR-246 was also assessed, and revealed that it had no appreciable stabilizing effect (Fig. 4F, Supplementary Fig. 3F). The same results were observed when applying APR-246 directly to cell lysate (Supplementary Fig. 3FG, H). Since APR-246 is metabolized to MQ, we reasoned that direct treatment of the cell lysate with APR-246 may not allow for this conversion. As such, we performed the experiment using MQ, revealing no effect on stability (Supplementary Fig. 3I, J). Other studies demonstrating the stabilizing effect of APR-246 have done so using the DBD rather than the whole protein [7, 10, 34]. Additionally, it is known that a certain number of wild-type subunits must be present for the tetrameric protein to restore wild-type function [35]. Thus, we posit that the dose of APR-246 needed to sufficiently restore wildtype function and overcome dominant negative effects of remaining p53 mutants is the major limitation in these studies.

### Redox homeostasis genes modulate response to APR-246 in a p53-dependent and p53-independent manner

**Fig. 5:**
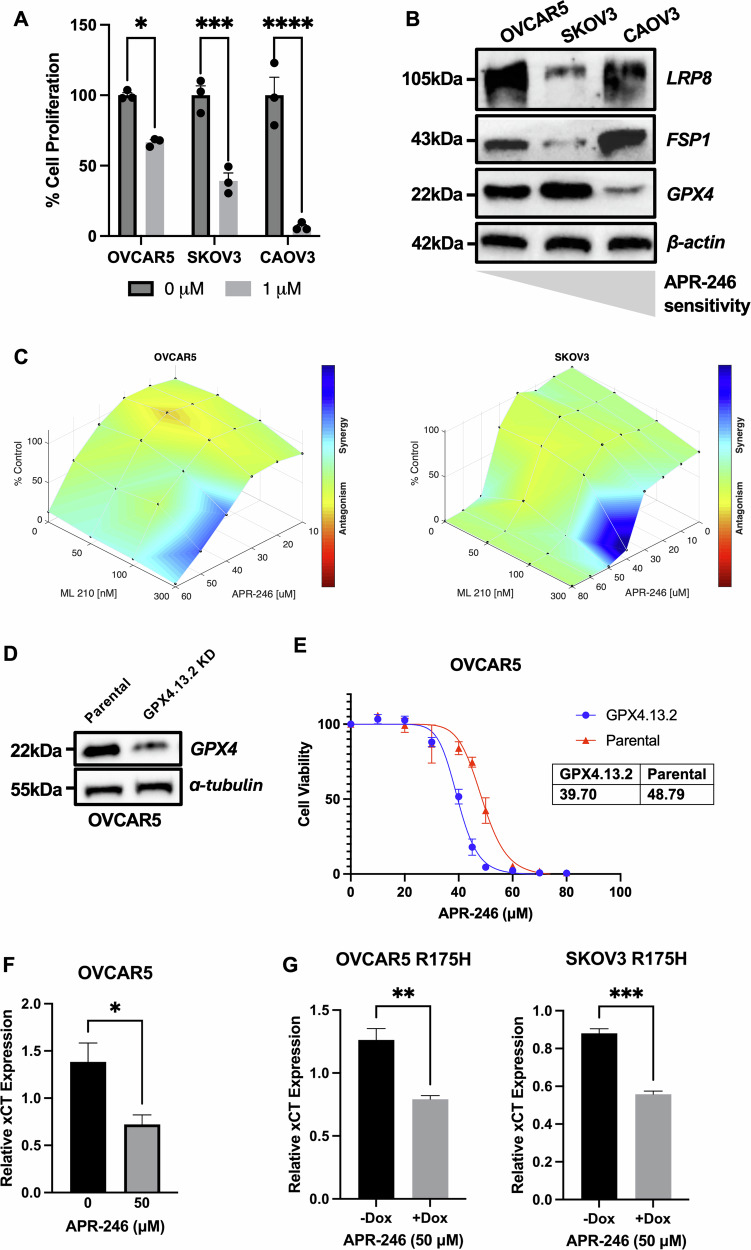
Redox homeostasis genes modulate response to APR-246 in a p53-dependent and p53-independent manner. **A** Colony formation assay of p53-null parental cells following treatment with APR-246. Cells were treated for 14-days prior to SRB staining. Data are shown as % cell proliferation relative to the vehicle-treated group (*n* = 3). Statistical analysis was performed using a 2-way ANOVA with multiple comparisons to the vehicle-treated group. **B** Western blot shows the differential expression of anti-ferroptotic proteins across parental cell lines. **C** Synergy assay between APR-246 and ML-210 in SKOV3 and OVCAR5 parental cell lines using the HAS model. **D** GPX4 knockdown in OVCAR5 cells 48 hours after siRNA transfection. **E** Dose-response of parental vs GPX4 siRNA knockdown OVCAR5 cells. Cells were treated for 72 hours prior to the Cell Titer Glo cell viability assay. Data are shown as % cell viability relative to the vehicle and fit to a sigmoidal 4PL model. **F** Gene expression data from RT-qPCR of xCT following 6 h treatment with APR-246 in p53-null OVCAR5. Data is shown as the mean + SEM (*n* = 3). Statistical analysis was performed using an unpaired t-test.G) Gene expression data from RT-qPCR of xCT following 6 h treatment with APR-246 in R175H-expressing OVCAR5 and SKOV3 cells. Data is shown as the mean + SEM (*n* = 3). Statistical analysis was performed using an unpaired t-test. *p < 0.05, **p < 0.005, ***p < 0.0005, ****p < 0.0001.

Previous studies have characterized APR-246 as a modulator of redox homeostasis and inducer of ROS through depletion of key antioxidants, resulting in both p53-dependent and -independent cell death pathways such as ferroptosis [26–29]. Indeed, dose response studies with APR-246 in the presence of known-antioxidant NAC demonstrates the complete ablation of the cytotoxic effect of APR-246 (Supplementary Fig. 4A). However, we submit that given APR-246’s known activity as a cystine-binding molecule it is likely being sequestered by the cystine made available by NAC, rendering these results, which have also been shown by others, as largely misleading [36, 37]. Given the known off-target effects of APR-246, we investigated whether the mutant-specific effects observed in OVCAR5 cells were attributable to intrinsic differences in redox homeostasis among the parental p53-null cell lines. We demonstrated that our p53-null cell line models exhibited differential intrinsic sensitivity to APR-246 in short-term cytotoxicity assays (Fig. 1B) as well as long-term clonogenic assays (Fig. 5A). These results indicate that parental OVCAR5 are less responsive to APR-246 than SKOV3, which are in turn less responsive than CAOV3. To understand why OVCAR5 is less responsive to APR-246, we assessed the expressions of key proteins regulating redox homeostasis and ferroptosis sensitivity. Western blot analysis of our three parental cell lines indicates that expression of anti-ferroptosis genes, GPX4, FSP1, and LRP8 is varied (Fig. 5B). We observe that OVCAR5 has modest to robust expression of all three genes while SKOV3 and CAOV3 each lack robust expression of at least one of the three genes (Fig. 5B). We noted that the expression of GPX4 seemed to correlate best with APR-246 sensitivity. Indeed, APR-246 was synergistic with GPX4 inhibitor, ML-210, in parental OVCAR5 and SKOV3 (Fig. 5C). Parallel studies in OVCAR5 expressing R175H showed that induction of p53 did not enhance the synergy between APR-246 and ML-210 (Supplementary Fig. 4B, C). Additionally, OVCAR5 with siRNA-mediated knockdown of *GPX4* were sensitized to APR-246 treatment as observed by the decrease in IC_50_ from ~50 μM to ~40 μM (Fig. 5D, E). In addition, we examined the role of glutathione transporter, xCT, in APR-246 treatment. We observed a downregulation in xCT expression upon treatment of p53-null OVCAR5 cells with APR-246 (Fig. 5F), consistent with the role of APR-246 as a stimulator of ROS. Further, induction of R175H expression under APR-246 treatment repressed xCT expression in both OVCAR5 and SKOV3 cells (Fig. 5G). Interestingly, induction of R175H alone in the absence of APR-246 did not significantly repress xCT expression (Supplementary Fig. 4D). Given the role of WT p53 as a known transcriptional repressor of xCT the data suggests that the suppression observed in the treated state (Fig. 5G) is due to the mutant-specific rescue effect of APR-246. This is further supported by our observation that expression of R175H in OVCAR5 does not sensitize cells to known ferroptosis inducer, Erastin (Supplementary Fig. 4E). Interestingly, while ferroptosis inhibitor, Ferrostatin, was able to fully rescue the cell death caused by Erastin treatment, it was unable to rescue cell death caused by APR-246 (Supplementary Fig. 4E). This may be reflective of the promiscuous nature of APR-246 as an inducer of cell death via various independent pathways. These observations support a model of APR-246 activity in which cell death is induced via various mechanisms in both p53-dependent and p53-independent manners.

## Discussion

APR-246 was identified in the monumental effort to create a pan-acting mutant p53 rescuing drug. Despite studies indicating that it had the potential to rescue various p53 missense mutants [38, 39], many studies, including this one, have suggested otherwise. There are many contradictory findings made in the literature, including the fact that APR-246, as a conformationally stabilizing compound, should primarily affect conformational p53 mutants. Despite this, some studies demonstrated p53-specific effects in known DNA-contact hotspot mutants such as R248Q [4, 40] and R273H [10]. In contrast, several studies reported that APR-246’s activity is independent of p53 status, whether WT, null, or missense mutated [41–45]. There is substantial evidence that APR-246’s effect on the redox system contributes considerably to its mechanism of action [20, 26, 46], and that this triggers ferroptosis [27–29] independent of p53 status [47].

We hypothesize that the major mechanism of action of APR-246 is its redox modulating activity, while the minor mechanism is its limited potential for p53 rescue. Due to the scale being overwhelmingly tipped towards redox modulation, identification of a dose of APR-246 that allows for observation of a p53-specific effect is challenging. We note that much of the literature performs experiments at doses that are likely to induce the non-p53-related effects of the drug in non-isogenic cell lines bearing different p53 status. As such, it becomes difficult to properly evaluate the impact that differences in redox sensitivity may have on determining response to APR-246. Our study addresses this by using an inducible system, which allows us to exclusively observe the extent of the p53-specific effect and suggests that even the prototypical conformational mutant R175H has a modest effect on response to APR-246. We hypothesize that this limited effect may also be attributable to a narrow dosing window within which a p53-specific effect may be observed. We know from structural studies that due to its tetrameric nature, p53 requires a certain number of WT subunits to maintain functionality [35], and that many pathogenic p53 variants exhibit a dominant negative effect. In the context of mutant p53 overexpression, which is often observed from pathogenic variants, the amount of APR-246 needed to restore the number of subunits necessary to overcome a dominant negative effect may very well surpass the threshold necessary to modulate the redox system.

As such, we hypothesize that a p53-specific rescue effect may only be observable under a specific set of conditions. These include a relatively redox/ferroptosis-resistant background, a narrow dosing window, and the presence of a rescuable conformational p53 mutation. We attribute the challenges of identifying APR-246 responsive mutants to these factors. Indeed, although our screen indicates a decrease in total cell count upon treatment, suggesting a cytotoxic effect, there was limited change to variant distribution, suggesting that there was a global decrease in the population rather than a specific sub-population of sensitive mutants. We also conclude that, due to the difficulty in observing a p53-specific effect, we could not determine any effect of the P72R SNP on p53 rescue by APR-246.

It is plausible that factors that play a role in redox homeostasis may play an impactful role in determining APR-246 sensitivity [48]. We demonstrate that genetic and pharmacological modulation of *GPX4* expression impacts APR-246 sensitivity. We also demonstrate that APR-246 modulates xCT expression in both a p53-dependent and -independent manner. This contributes to the larger discussion around the potential promise of APR-246 as an inducer of ferroptosis rather than a p53-rescue drug in a clinical setting [28], [48]. In conclusion, we suggest that APR-246 exerts its cytotoxic effect through various cell death pathways. Furthermore, we posit that cancer cells that are intrinsically resistant to redox modulation facilitate a cellular state in which observations of the p53 mutant-specific rescue effects of APR-246 can be observed.

## Methods

### Cell lines and cell culture

SKOV3 cells obtained from Sigma-Aldrich were maintained in McCoy’s (Gibco) containing 10% FBS (Gibco) and 1% Antibiotic-Antimycotic (Gibco). H1299 cells were obtained from ATCC and were maintained in McCoy’s (Gibco) containing 10% FBS (Gibco) and 1% Antibiotic-Antimycotic (Gibco). OVCAR5 cells obtained from the NCI DCTD Tumor Repository and CAOV3 cells obtained from ATCC were maintained in RPMI (Gibco) containing 10% FBS (Gibco) and 1% Antibiotic-Antimycotic (Gibco). H293T cells were maintained in DMEM (Gibco) containing 10% FBS (Gibco) and 1% Antibiotic-Antimycotic (Gibco). All mutant cell lines generated in any of the above cells were cultured and maintained in the same culture conditions as the parental cells.

### TP53 MITE Library Amplification

*TP53* MITE library was a gift from William Hahn and David Root (Addgene #113569; http://n2t.net/addgene:113569; RRID:Addgene_113569) (Watertown, MA, USA) and amplified according to the plasmid DNA Library Amplification protocol using 10-beta Electrocompetent *E. coli* (NEB, Ipswich, MA, USA). We obtained a representation of >3000 bacterial colonies per variant. Plasmid DNA was extracted from the harvested colonies using the ZymoPure II Plasmid Maxiprep Kit (Zymo Research, Irvine, CA, USA).

### Lentivirus production

Lentivirus was produced in HEK-293T cells which were transfected using Lipofectamine 3000 (Thermo Fisher Scientific, Waltham, MA, USA) with the plasmid of interest, a packaging plasmid psPAX2, a gift from Didier Trono (Addgene plasmid # 12260; http://n2t.net/addgene:12260; RRID:Addgene_12260), and an envelope plasmid pMD2.G, a gift from Didier Trono (Addgene plasmid # 12259; http://n2t.net/addgene:12259; RRID:Addgene_12259). Media was harvested at 24 and 48 hours post-transfection. Viral titer was determined using the qPCR Lentivirus Titer Kit (Applied Biological Materials, Richmond, BC).

### TP53 MITE library screen with APR-246

Appropriate cell lines were infected with the *TP53* MITE library viral supernatant supplemented with polybrene infection reagent (5μg/mL) and selected with puromycin (1–5μg /mL) for 10–14 days. Cells were then expanded and seeded at a density of 3E6 cells/10 cm dish. Cells were treated with the appropriate dose of APR-246 24 hours after seeding. Cells were harvested after 48 hours of treatment and subjected to genomic DNA (gDNA) extraction using QIAamp DNA Blood Midi Kits (Qiagen, Germantown, MD, USA).

### TP53 coding DNA sequence (CDS) purification from gDNA

TP53 CDS was amplified as previously described [19], with the exception of the last purification step with AMPure XP (Beckman Coulter, Indianapolis, IN, USA).

### Illumina sequencing

Library preparation was performed according to the Illumina Nextera XT protocol. For each sample, we set up 3 reactions, each with 1 ng of purified CDS DNA. Each reaction was uniquely indexed with i7/i5 indices. Samples were purified with the AMPure XP kit (Beckman Coulter). Samples were QC’ed, pooled, and sequenced by Novogene (Sacramento, CA, USA) using the Illumina HiSeq PE150 platform.

### Identification of candidate mutants that respond to APR246

Fastq sequencing reads were trimmed and mapped to TP53 mRNA sequence (NM_000546) using BWA MEM, and resulting BAM files were processed using GATK AnalyzeSaturationMutagenesis to generate read counts for each codon variant. Reference sequence and example script are available at the following link: https://osf.io/2dmj4/overview?view_only=e4df1dbd3af846dc8c5bcbcf5bb53849. The counts were converted to Z-scores, and variants with <= -2 Z-scores were considered as candidate variants that respond to APR246.

### Selection of candidate mutants for functional validation

Candidate variants were further screened for their prevalence in the TCGA patient population. Candidate variants with the highest prevalence in the patient population were selected for functional validation.

### Candidate mutant cloning

Variants selected for functional validation were cloned into a tetracycline-inducible lentiviral plasmid using the NEBBuilder cloning kit, and the viral supernatant generated in HEK293T cells was used to transduce the ovarian cancer cell line OVCAR5. Stably transduced cells were selected with 0.5μg/mL puromycin.

### Colony formation assays

2000 cells were seeded in 6-well plates. The medium, containing appropriate treatment, was changed every 2–3 days to allow colonies to form. At the end of the experiments (14 days), SRB assay was performed. Cells were fixed with 10% Trichloroacetic acid (TCA) and stained with Sulphorhodamine (SRB) dye. Colonies were imaged with the Molecular Imager Gel Doc XR System (Bio-Rad), followed by solubilization in Tris Buffer and absorbance readings at 510 nm with a plate reader (Molecular Devices, San Jose, CA, USA). The statistical analysis and graphs of colonies were generated in GraphPad Prism version 10.2.3.

### Flow cytometry experiments

p53 mutant cells were transduced and selected with puromycin. To assess p53 expression, flow cytometric analysis was carried out on live cells, and GFP expression was monitored using the FITC channel on a BD LSRII. GFP/FITC was used as a surrogate marker for p53 expression. To assess cell cycle distribution, flow cytometric analysis was carried out on fixed cells and stained with DAPI at 10 μg/mL. GFP expression was monitored using the FITC channel, and DAPI was monitored using the UV channel on a BD LSRII. Cell cycle analysis data was generated in FlowJo version 10.10.0.

### Antibodies and compounds

Secondary antibodies, horse anti-mouse IgG-HRP antibody (7076S) and goat anti-rabbit IgG-HRP antibody (7074S) were purchased from Cell Signaling Technologies (Danvers, MA, USA). P53 Antibody (DO-1) was purchased from Santa Cruz Biotechnology (sc-126) (Dallas, TX, USA). AMID/AIFM2/FSP1 Antibody (B-6) was purchased from Santa Cruz Biotechnology (sc-377120). xCT/SLC7A11 Antibody was purchased from Cell Signaling Technologies (12691T). LRP8 Antibody was purchased from Invitrogen (MA5-53502) (Waltham, MA, USA). P21 Antibody was purchased from Invitrogen (MA5-14949). β -actin Antibody (C4) HRP was purchased from Santa Cruz Biotechnology (sc-47778 HRP). α-tubulin antibody was purchased from DSHB (12G10) (University of Iowa, Iowa City, IA, USA). Eprenetapopt/APR-246/PRIMA-1^MET^ was purchased from Selleckchem (S7724) (Houston, TX, USA). APR-246 stock solutions were made with DMSO at 10 mM and stored at −80 °C. ML 210 was purchased from Selleckchem (S7088). ML 210 stock solutions were made fresh with DMSO at 5 mM and stored at -80°C.

### Immunoblotting

Cells were washed with PBS at the end of treatments if applicable, and then lysed with an appropriate volume of 2X Laemmli sample buffer (Bio-Rad Laboratories, Hercules, CA, USA)

with 5% β-mercaptoethanol (Bio-Rad Laboratories). The cell lysates were then boiled at 100 °C for 10 minutes before use. Lysate was subjected to SDS-polyacrylamide gel electrophoresis and electroblotted onto PVDF membranes. After blocking with 5% nonfat dry milk in TBS-Tween for 1–2 h at room temperature, blots were incubated with appropriate primary antibodies overnight at 4 °C. Blots were then washed and incubated with appropriate horseradish peroxidase (HRP)-conjugated secondary antibodies for 1 h, and protein bands were visualized using a chemiluminescence kit (Thermo Scientific, Rockford, IL, USA). Next, blots were stripped, blocked, and re-probed for other proteins of interest or β-actin. The expression level of each protein was normalized to the level of β-actin. Densitometric analysis was performed with Image J software (NIH).

### Immunofluorescence

Cells were washed with PBS at the end of treatments, if applicable, and then fixed with 4% paraformaldehyde for 15 minutes. Cells were blocked and lysed for 1 hour with a solution of 1.5% BSA and 0.3% Triton X-100 in TBS-Tween. Cells were then incubated with the appropriate primary antibody overnight at 4 °C. Cells were incubated with appropriate secondary antibody conjugated with DyLight-488 or 650 (Invitrogen) for 1 hour. Finally, cells were incubated with DAPI prior to imaging with the ImageXpress Pico Automated Cell Imaging System (Molecular Devices).

### CCK8 cytotoxicity assay

Cells were plated into 96-well plates (2 × 10^3^ cells/100μL/well) and cultured in growth medium overnight. The next day, cells were treated with appropriate concentrations of APR-246. 72 hours later, cell viability was assessed using CCK8 (GlpBio, Montclair, CA, USA). Dose-response curves were fitted, and the IC50 for each drug was determined using GraphPad Prism 10.2.3. All curves were constrained with 100% on top.

### siRNA transfections

*GPX4*-specific siRNAs were purchased from IDT (hs.R1.GPX4.13.2, Coralville, IA, USA). 3 ×106 cells/well were seeded in a 10 cm dish and incubated at 37 °C overnight. The next day, 40 nM of each siRNA was transfected into the cells with Lipofectamine 3000 (Invitrogen) according to the manufacturer’s instructions. 24 hours after siRNA transfection, cells were trypsinized and seeded in 96-well plates and 35 mm dishes alongside parental cells and kept overnight. Approximately 72 hours after siRNA transfection, cells were either collected for western blot or treated with APR-246 for 72 hours before the Cell Titer Glo cell viability assay.

Supplementary information is available at Cell Death Discovery’s website https://www.nature.com/cddiscovery/.

### Ethics

This study did not involve human participants, human data, or human tissue, and did not require ethical approval.

## Supplementary information

Supplementary Figures

Original Western Blot Images

## Data Availability

Reference sequence, example script, and raw count data from the screening study is available at the following repository: https://osf.io/2dmj4/overview?view_only=e4df1dbd3af846dc8c5bcbcf5bb53849.

## References

[1] Zhang C, Liu J, Xu D, Zhang T, Hu W, Feng Z Gain-of-function mutant p53 in cancer progression and therapy. Lu H, editor. J Mol Cell Biol. 2020;12:674–87. 10.1093/jmcb/mjaa040 PMC7749743 32722796

[2] Mendiratta G, Ke E, Aziz M, Liarakos D, Tong M, Stites EC. Cancer gene mutation frequencies for the U.S. population. Nat Commun. 2021;12:5961. 34645806 10.1038/s41467-021-26213-y PMC8514428

[3] Yang-Hartwich Y, Soteras MG, Lin ZP, Holmberg J, Sumi N, Craveiro V, et al. p53 protein aggregation promotes platinum resistance in ovarian cancer. Oncogene. 2015;34:3605–16. 25263447 10.1038/onc.2014.296

[4] Xi Y, Guo Y, Qiu S, Lv F, Deng Y, Xie J, et al. Trends in gynaecologic cancer mortality and the impact of the COVID-19 pandemic in the United States. Infect Agent Cancer. 2024;19:4. 38378712 10.1186/s13027-024-00567-6 PMC10880335

[5] Butler JS, Loh SN. Structure, function, and aggregation of the zinc-free form of the p53 DNA binding domain. Biochemistry. 2003;42:2396–403. 12600206 10.1021/bi026635n

[6] Bullock AN, Henckel J, DeDecker BS, Johnson CM, Nikolova PV, Proctor MR, et al. Thermodynamic stability of wild-type and mutant p53 core domain. Proc Natl Acad Sci USA. 1997;94:14338–42. 9405613 10.1073/pnas.94.26.14338 PMC24967

[7] Khadiullina R, Mirgayazova R, Davletshin D, Khusainova E, Chasov V, Bulatov E. Assessment of thermal stability of mutant p53 Proteins via Differential Scanning Fluorimetry. Life. 2022;13:31. 36675980 10.3390/life13010031 PMC9862671

[8] Nishikawa S, Iwakuma T. Drugs Targeting p53 mutations with FDA approval and in clinical trials. Cancers. 2023;15:429. 36672377 10.3390/cancers15020429 PMC9856662

[9] Sallman DA, DeZern AE, Garcia-Manero G, Steensma DP, Roboz GJ, Sekeres MA, et al. Eprenetapopt (APR-246) and Azacitidine in TP53-Mutant Myelodysplastic Syndromes. J Clin Oncol J Am Soc Clin Oncol. 2021;39:1584–94. 10.1200/JCO.20.02341 PMC8099410 33449813

[10] Zhang Q, Bykov VJN, Wiman KG, Zawacka-Pankau J. APR-246 reactivates mutant p53 by targeting cysteines 124 and 277. Cell Death Dis. 2018;9:439. 29670092 10.1038/s41419-018-0463-7 PMC5906465

[11] Ceder S, Eriksson SE, Cheteh EH, Dawar S, Corrales Benitez M, Bykov VJN, et al. A thiol-bound drug reservoir enhances APR-246-induced mutant p53 tumor cell death. EMBO Mol Med. 2021;13:e10852. 33314700 10.15252/emmm.201910852 PMC7863383

[12] Elayapillai S, Ramraj S, Benbrook DM, Bieniasz M, Wang L, Pathuri G, et al. Potential and mechanism of mebendazole for treatment and maintenance of ovarian cancer. Gynecol Oncol. 2021;160:302–11. 33131904 10.1016/j.ygyno.2020.10.010 PMC8820236

[13] Hoang T, Sutera P, Nguyen T, Chang J, Jagtap S, Song Y, et al. TP53 structure-function relationships in metastatic castrate-sensitive prostate cancer and the impact of APR-246 treatment. Prostate. 2024;84:87–99. 37812042 10.1002/pros.24629

[14] Baugh EH, Ke H, Levine AJ, Bonneau RA, Chan CS. Why are there hotspot mutations in the TP53 gene in human cancers?. Cell Death Differ. 2018;25:154–60. 29099487 10.1038/cdd.2017.180 PMC5729536

[15] De Souza C, Madden J, Koestler DC, Minn D, Montoya DJ, Minn K, et al. Effect of the p53 P72R polymorphism on mutant *TP53* Allele selection in human cancer. JNCI J Natl Cancer Inst. 2021;113:1246–57. 33555293 10.1093/jnci/djab019 PMC8633460

[16] Kung CP, Liu Q, Murphy ME. The codon 72 polymorphism of p53 influences cell fate following nutrient deprivation. Cancer Biol Ther. 2017;18:484–91. 28475405 10.1080/15384047.2017.1323595 PMC5639853

[17] Dumont P, Leu JIJ, Della Pietra AC, George DL, Murphy M. The codon 72 polymorphic variants of p53 have markedly different apoptotic potential. Nat Genet. 2003;33:357–65. 12567188 10.1038/ng1093

[18] Barnoud T, Parris JLD, Murphy ME. Common genetic variants in the TP53 pathway and their impact on cancer. J Mol Cell Biol. 2019;11:578–85. 31152665 10.1093/jmcb/mjz052 PMC6736421

[19] Giacomelli AO, Yang X, Lintner RE, McFarland JM, Duby M, Kim J, et al. Mutational processes shape the landscape of TP53 mutations in human cancer. Nat Genet. 2018;50:1381–7. 30224644 10.1038/s41588-018-0204-y PMC6168352

[20] Bykov VJN, Zhang Q, Zhang M, Ceder S, Abrahmsen L, Wiman KG targeting of mutant p53 and the Cellular Redox Balance by APR-246 as a strategy for efficient cancer therapy. Front Oncol [Internet]. 2016 [cited 2023 June 5];6. Available from: https://www.frontiersin.org/articles/10.3389/fonc.2016.00021. 10.3389/fonc.2016.00021 PMC4737887 26870698

[21] Whibley C, Pharoah PDP, Hollstein M. p53 polymorphisms: cancer implications. Nat Rev Cancer. 2009;9:95–107. 19165225 10.1038/nrc2584

[22] Thomas M, Kalita A, Labrecque S, Pim D, Banks L, Matlashewski G. Two polymorphic variants of wild-type p53 differ biochemically and biologically. Mol Cell Biol. 1999;19:1092–100. 9891044 10.1128/mcb.19.2.1092 PMC116039

[23] Pim D, Banks L. p53 polymorphic variants at codon 72 exert different effects on cell cycle progression. Int J Cancer. 2004;108:196–9. 14639602 10.1002/ijc.11548

[24] Frank AK, Leu JIJ, Zhou Y, Devarajan K, Nedelko T, Klein-Szanto A, et al. The Codon 72 Polymorphism of p53 Regulates Interaction with NF-κB and Transactivation of Genes Involved in Immunity and Inflammation. Mol Cell Biol. 2011;31:1201–13. 21245379 10.1128/MCB.01136-10 PMC3067895

[25] Marin MC, Jost CA, Brooks LA, Irwin MS, O’Nions J, Tidy JA, et al. A common polymorphism acts as an intragenic modifier of mutant p53 behaviour. Nat Genet. 2000;25:47–54. 10802655 10.1038/75586

[26] Synnott NC, Madden SF, Bykov VJN, Crown J, Wiman KG, Duffy MJ. The mutant p53-targeting compound APR-246 induces ROS-modulating genes in breast cancer cells. Transl Oncol. 2018;11:1343–9. 30196236 10.1016/j.tranon.2018.08.009 PMC6132178

[27] Birsen R, Larrue C, Decroocq J, Johnson N, Guiraud N, Gotanegre M, et al. APR-246 induces early cell death by ferroptosis in acute myeloid leukemia. Haematologica. 2021;107:403–16. 10.3324/haematol.2020.259531 PMC8804578 33406814

[28] Fujihara KM, Zhang BZ, Jackson TD, Ogunkola MO, Nijagal B, Milne JV, et al. Eprenetapopt triggers ferroptosis, inhibits NFS1 cysteine desulfurase, and synergizes with serine and glycine dietary restriction. Sci Adv. 2022;8:eabm9427. 36103522 10.1126/sciadv.abm9427 PMC9473576

[29] Hong Y, Ren T, Wang X, Liu X, Fei Y, Meng S, et al. APR-246 triggers ferritinophagy and ferroptosis of diffuse large B-cell lymphoma cells with distinct TP53 mutations. Leukemia. 2022;36:2269–80. 35835991 10.1038/s41375-022-01634-w

[30] De Souza C, Madden JA, Minn D, Kumar VE, Montoya DJ, Nambiar R, et al. The P72R polymorphism in R248Q/W p53 mutants modifies the mutant effect on epithelial to mesenchymal transition phenotype and cell invasion via CXCL1 expression. Int J Mol Sci. 2020;21:8025. 33126568 10.3390/ijms21218025 PMC7662892

[31] Schneider-Stock R, Boltze C, Peters B, Szibor R, Landt O, Meyer F, et al. Selective loss of codon 72 proline p53 and frequent mutational inactivation of the retained arginine allele in colorectal cancer. Neoplasia N Y N. 2004;6:529–35. 10.1593/neo.04178 PMC1531656 15548361

[32] Papadakis ED, Soulitzis N, Spandidos DA. Association of p53 codon 72 polymorphism with advanced lung cancer: the Arg allele is preferentially retained in tumours arising in Arg/Pro germline heterozygotes. Br J Cancer. 2002;87:1013–8. 12434294 10.1038/sj.bjc.6600595 PMC2364333

[33] Lambert JMR, Gorzov P, Veprintsev DB, Söderqvist M, Segerbäck D, Bergman J, et al. PRIMA-1 reactivates mutant p53 by covalent binding to the core domain. Cancer Cell. 2009;15:376–88. 19411067 10.1016/j.ccr.2009.03.003

[34] Chan WM, Siu WY, Lau A, Poon RYC. How many mutant p53 Molecules Are Needed To Inactivate a Tetramer?. Mol Cell Biol. 2004;24:3536–51. 15060172 10.1128/MCB.24.8.3536-3551.2004 PMC381690

[35] Liu DS, Duong CP, Haupt S, Montgomery KG, House CM, Azar WJ, et al. Inhibiting the system xC−/glutathione axis selectively targets cancers with mutant-p53 accumulation. Nat Commun. 2017;8:14844. 28348409 10.1038/ncomms14844 PMC5379068

[36] Yoshikawa N, Kajiyama H, Nakamura K, Utsumi F, Niimi K, Mitsui H, et al. PRIMA-1MET induces apoptosis through accumulation of intracellular reactive oxygen species irrespective of p53 status and chemo-sensitivity in epithelial ovarian cancer cells. Oncol Rep. 2016;35:2543–52. 26986846 10.3892/or.2016.4653 PMC4811399

[37] Degtjarik O, Golovenko D, Diskin-Posner Y, Abrahmsén L, Rozenberg H, Shakked Z. Structural basis of reactivation of oncogenic p53 mutants by a small molecule: methylene quinuclidinone (MQ). Nat Commun. 2021;12:7057. 34862374 10.1038/s41467-021-27142-6 PMC8642532

[38] Kobayashi T, Makino T, Yamashita K, Saito T, Tanaka K, Takahashi T, et al. APR-246 induces apoptosis and enhances chemo-sensitivity via activation of ROS and TAp73-Noxa signal in oesophageal squamous cell cancer with TP53 missense mutation. Br J Cancer. 2021;125:1523–32. 34599296 10.1038/s41416-021-01561-0 PMC8608903

[39] Lee D, Jeong HS, Hwang SY, Lee YG, Kang YJ. ABCB1 confers resistance to carboplatin by accumulating stem-like cells in the G2/M phase of the cell cycle in p53null ovarian cancer. Cell Death Discov. 2025;11:132. 40175339 10.1038/s41420-025-02435-7 PMC11965561

[40] Li X, Zhou J, Tang NX, Chai Y, Zhou M, Gao A, et al. Molecular mechanisms of synergistic effect of PRIMA -1^met^ and Oxaliplatin in colorectal cancer with different p53status. Cancer Med. 2025;14:e70530. 39757707 10.1002/cam4.70530 PMC11702439

[41] Xiao S, Shi F, Song H, Cui J, Zheng D, Zhang H, et al. Characterization of the generic mutant p53-rescue compounds in a broad range of assays. Cancer Cell. 2024;42:325–7. 38402608 10.1016/j.ccell.2024.01.008

[42] Mohammed I, Alhammer AH, Arif IS. The p53 reactivator PRIMA-1MET synergises with 5-fluorouracil to induce apoptosis in pancreatic cancer cells. Invest N Drugs. 2023;41:587–95. 10.1007/s10637-023-01380-5 37402008

[43] Smith LE, Padilla JL, Licor A, Steinkamp MP, Lagutina IV, Guo Y, et al. Novel p53 reactivators that are synergistic with olaparib for the treatment of gynecologic cancers with mutant p53. Transl Oncol. 2025;61:102522. 40915175 10.1016/j.tranon.2025.102522 PMC12450570

[44] Michels J, Venkatesh D, Liu C, Budhu S, Zhong H, George MM, et al. APR-246 increases tumor antigenicity independent of p53. Life Sci Alliance. 2024;7:e202301999. 37891002 10.26508/lsa.202301999 PMC10610029

[45] Peng X, Zhang MQZ, Conserva F, Hosny G, Selivanova G, Bykov VJN, et al. APR-246/PRIMA-1MET inhibits thioredoxin reductase 1 and converts the enzyme to a dedicated NADPH oxidase. Cell Death Dis. 2013;4:e881. 24157875 10.1038/cddis.2013.417 PMC3920950

[46] Liu Y, Gu W. p53 in ferroptosis regulation: the new weapon for the old guardian. Cell Death Differ. 2022;29:895–910. 35087226 10.1038/s41418-022-00943-y PMC9091200

[47] Fujihara KM, Corrales Benitez M, Cabalag CS, Zhang BZ, Ko HS, Liu DS, et al. SLC7A11 is a superior determinant of APR-246 (Eprenetapopt) response than *TP53* mutation status. Mol Cancer Ther. 2021;20:1858–67. 34315763 10.1158/1535-7163.MCT-21-0067

[48] Magri J, Gasparetto A, Conti L, Calautti E, Cossu C, Ruiu R, et al. Tumor-associated antigen xCT and mutant-p53 as molecular targets for new combinatorial antitumor strategies. Cells. 2021;10:108. 33430127 10.3390/cells10010108 PMC7827209

