# Supplementary material for: Unbiased assessment of APR-246 responsive p53 mutants in ovarian cancer
Source: Cell Death Discov. 2026 May 19;12:297. doi: 10.1038/s41420-026-03152-5 (PMC13354765; doi:10.1038/s41420-026-03152-5)
Supplement: Supplementary file 1 — Supplementary Figures [file 41420_2026_3152_MOESM1_ESM.pdf]

A

Percentage of variants with zero representation			
	OVCAR5	SKOV3	Date of Screen
**Plasmid Input**	8	8	
**Control Cells**	52	50	
APR-246 (µM)	51	64	April-23
	51	64	
	51	49	May-23
	51	50	

B

TP53 Hotspot Mutant % Representation		
**Hotspot Mutant**	**SKOV3**	**OVCAR5**
**175H**	0.49	0.55
**245S**	0.06	0.07
**248Q**	0.05	0.07
**248W**	0.03	0.04
**249S**	0.03	0.04
**273C**	0.17	0.08
**273H**	0.02	0.02
**282H**	0	0
**282W**	0.03	0.04

C

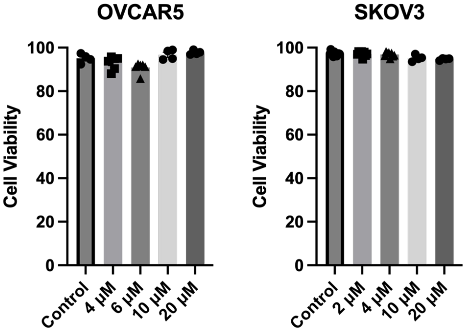

D

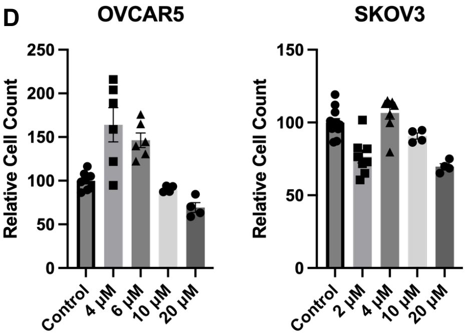

E

R175H *TP53 Enrichment Score*				
	APR-246 (µM)			
**OVCAR5**	-0.23	0.02	-0.42	-0.40
**SKOV3**	-	0.81	0.83	0.17

Supplementary Figure 1. A) Percentage of variants with zero representation in respective treatment groups. **B)** Average representation of individual hot-spot mutants in the control, untreated, populations for each respective cell line. **C)** Average cell viability (%) at time of cell harvest for each respective treatment group according to Countess 3 Cell Counter (Invitrogen). **D)** Average total cell count at time of cell harvest for each respective treatment group according to Countess. **E)** Average representation of R175H variant in the treatment populations for each respective cell line.

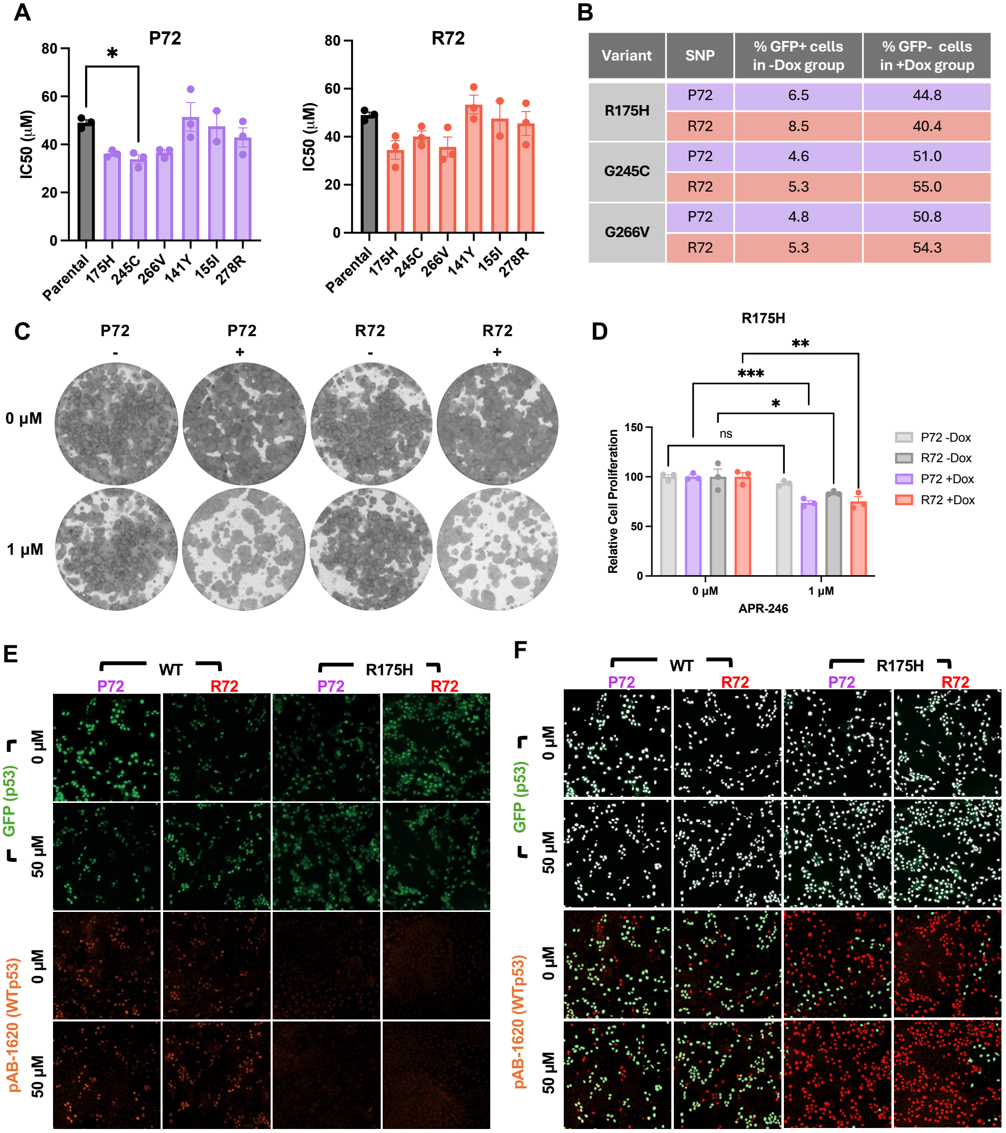

Variant	SNP	% GFP+ cells in -Dox group	% GFP- cells in +Dox group
R175H	P72	6.5	44.8
	R72	8.5	40.4
G245C	P72	4.6	51.0
	R72	5.3	55.0
G266V	P72	4.8	50.8
	R72	5.3	54.3

Supplementary Figure 2. A) IC50 values for each candidate variant under Tet- on conditions compared to p53-null parental cells. Mean IC50 + SEM (n=3*)**. **B)** Flow cytometry analysis of GFP-positive and negative (p53-positive and negative) cells under Tet-on and Tet-off conditions. **C)** Representative images of colony formation assay demonstrating a p53-specific effect of APR-246 in R175H-expressing OVCAR5, quantified in Figure 3D. **D)** Replicate of the experiment described in Figure 3D/Supplementary Figure 2C. **E)** Representative images of immunofluorescence assay of WT and R175H- expressing OVCAR5 cells following treatment with APR-246, quantified in Figure 3E. **F)** Representative images of cell scoring of the immunofluorescence assay quantified in Figure 3E. **Statistical analysis was performed using a 2-way ANOVA with multiple comparisons between variant and parental groups. ns = non-significant, *$p<0.05$, **$p<0.005$, ***$p<0.0005$. *(T155I n=2).

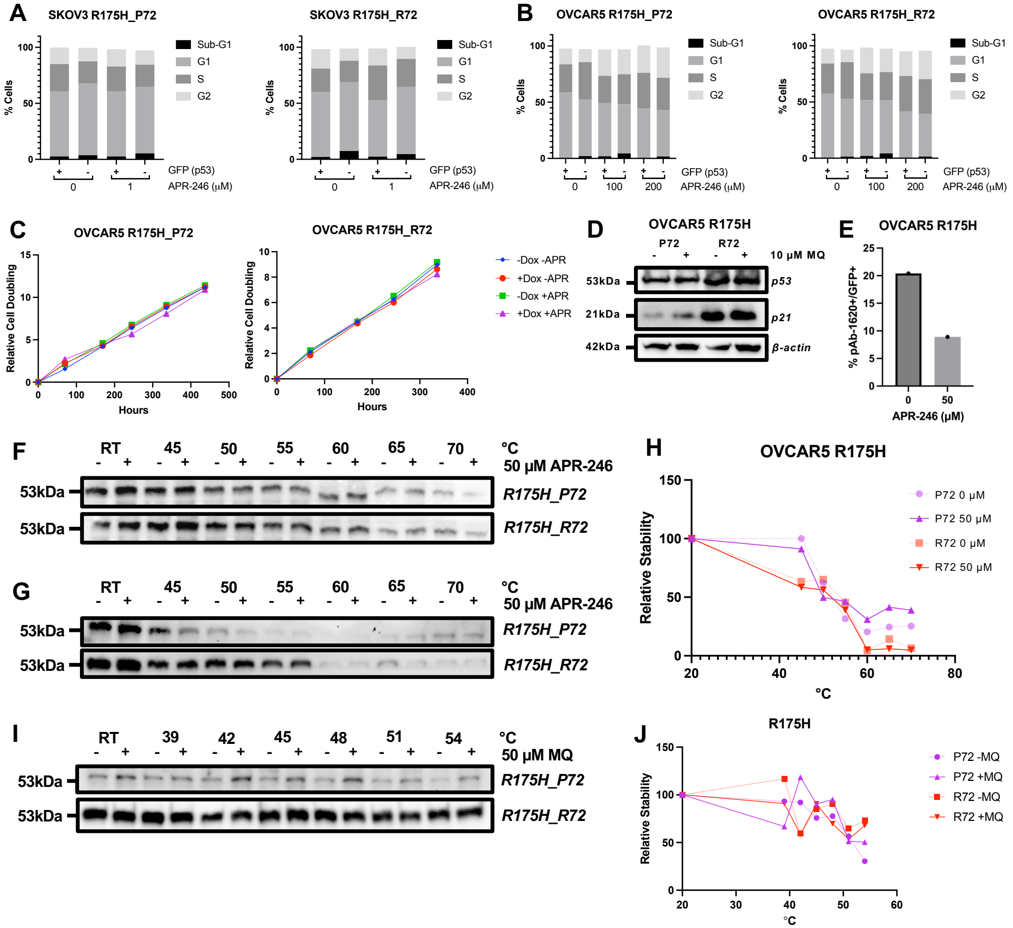

Supplementary Figure 3. A) Cell cycle analysis via flow cytometry. Cells were treated for 48 hours prior to processing. **B)** Cell cycle analysis via flow cytometry. Cells were treated for 4 hours prior to processing. **C)** Cell doubling assay assessing the cell growth dynamics of R175H cells with the P72 and R72 SNP under Tet-on/off and APR-246-treated/untreated conditions. **D)** Western blot of R175H-expressing OVCAR5 following 24h treatment with MQ. **E)** Percentage of R175H expressing cells that are recognized by the WT p53-recognizing pAb-1620 following 6h treatment with APR-246 as analyzed by flow cytometry. **F)** Western blot of CETSA assay to assess protein stability of R175H following in-cell treatment with APR-246 for 4h, quantified in Figure 4F. **G)** Western blot of CETSA assay to assess protein stability of R175H following lysate treatment with APR-246 for 2h, quantified in Supplementary Figure 3F. **H)** Quantification of Supplementary Figure 3E. **I)** Western blot of CETSA assay to assess protein stability of R175H following lysate treatment with MQ for 2hr, quantified in Supplementary Figure 3H. **J)** Quantification of Supplementary Figure 3G.

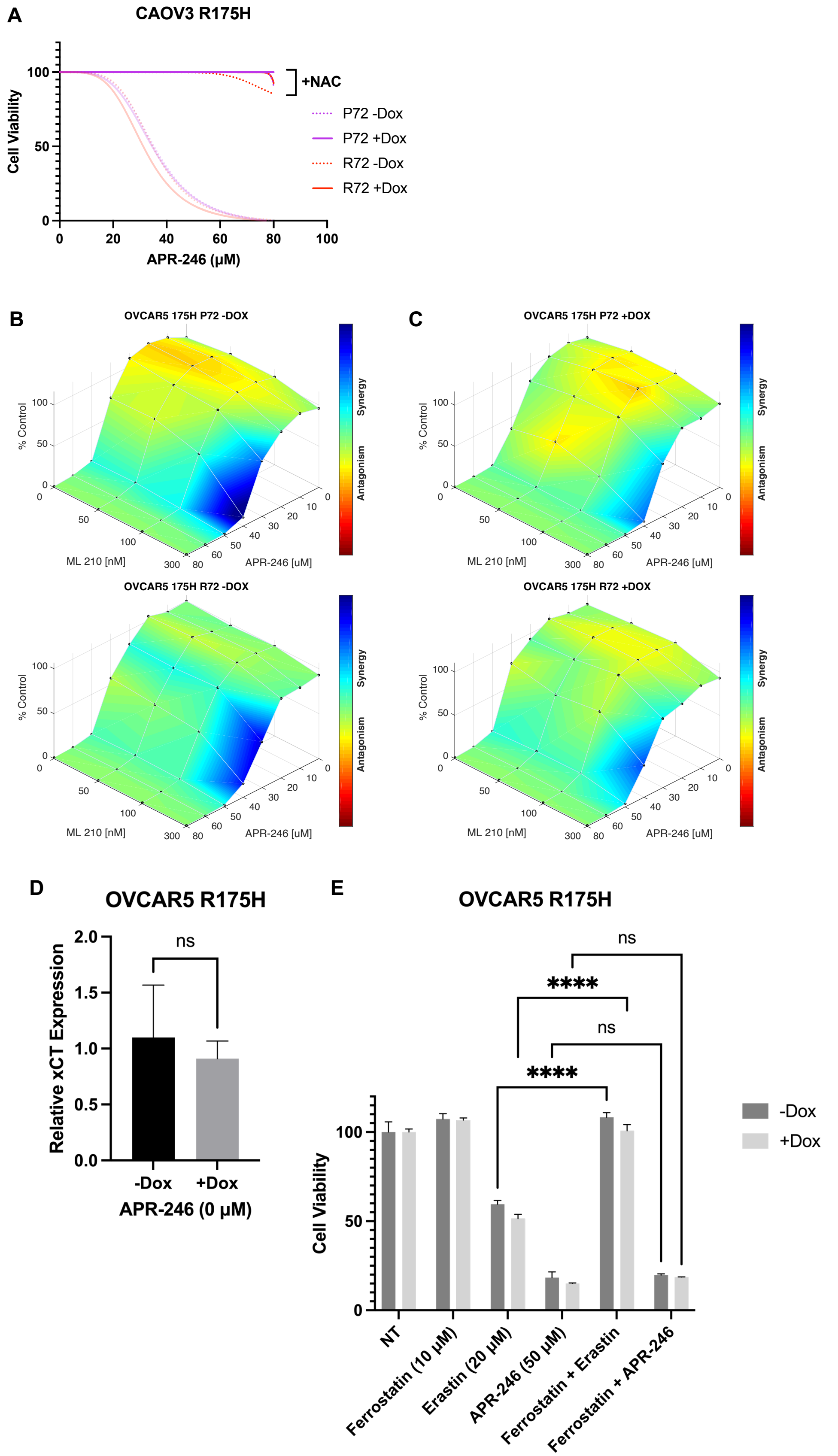

Supplementary Figure 4. A) Dose response of R175H-expressing CAOV3 treated with APR-246 in the absence and presence of NAC under Tet-on/off conditions. **B)** Synergy assay between APR-246 and ML-210 in R175H- expressing OVCAR5 cells under Tet-off conditions using the HAS model. **C)** Synergy assay between APR-246 and ML-210 in R175H-expressing OVCAR5 cells under Tet-on conditions using the HAS model**. D)** Gene expression data from RT-qPCR of xCT following induction of R175H expression in OVCAR5 cells. Data is shown as the mean + SEM (n=3). Statistical analysis was performed using an unpaired t-test. **E)** Cell viability assay in R175H-expressing OVCAR5 under Tet-on and Tet-off conditions. Cells were treated for 72hrs and cells treated with a combination of agents were pre-treated with Ferrostatin for 2hrs. Data is shown as the mean + SEM (n=6). Statistical analysis was performed using a 2-way ANOVA with multiple comparisons.
