# Supplementary material for: Unbiased assessment of APR-246 responsive p53 mutants in ovarian cancer
Source: Cell Death Discov. 2026 May 19;12:297. doi: 10.1038/s41420-026-03152-5 (PMC13354765; doi:10.1038/s41420-026-03152-5)

Figure 5B

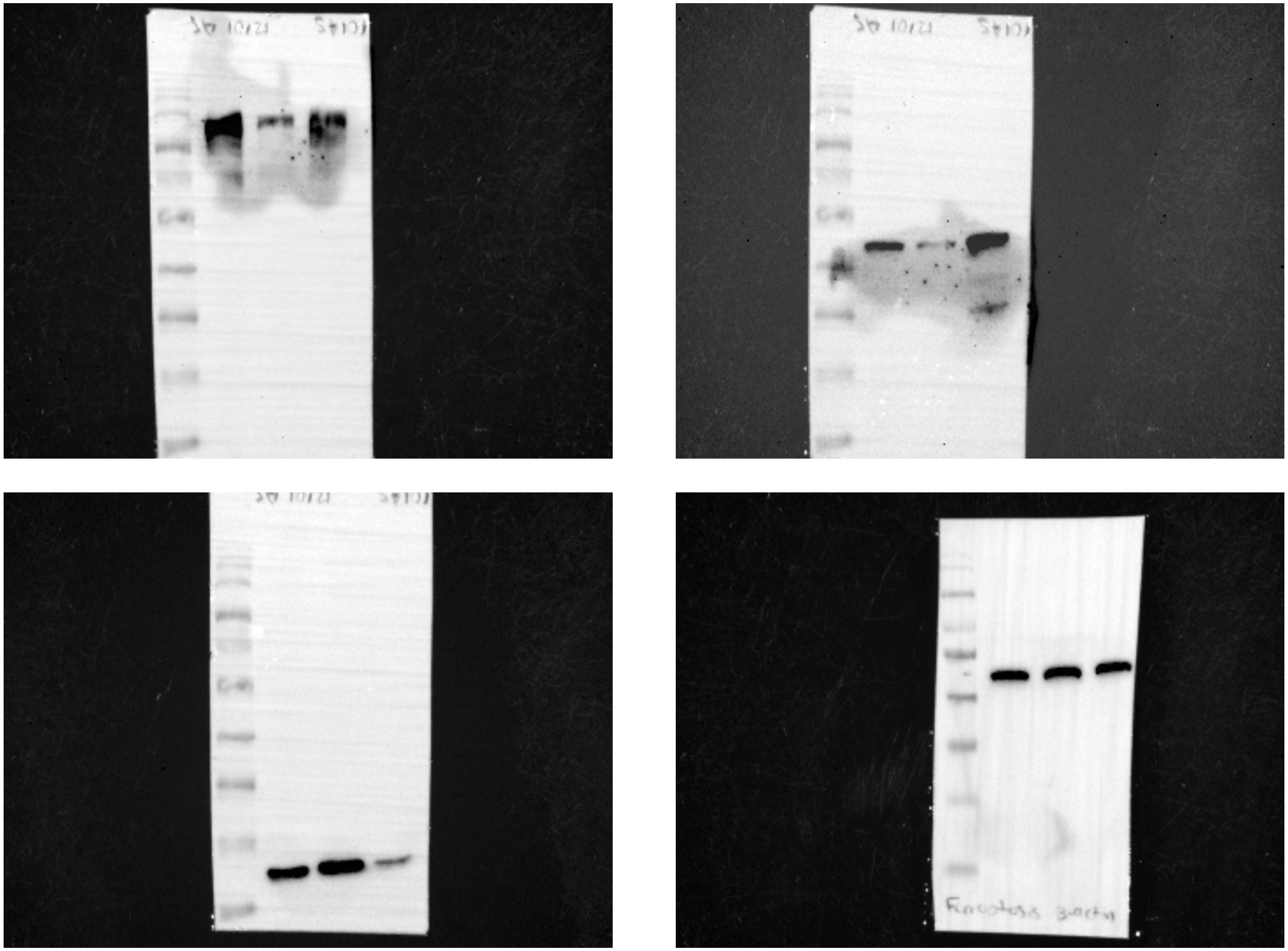

Figure 5D

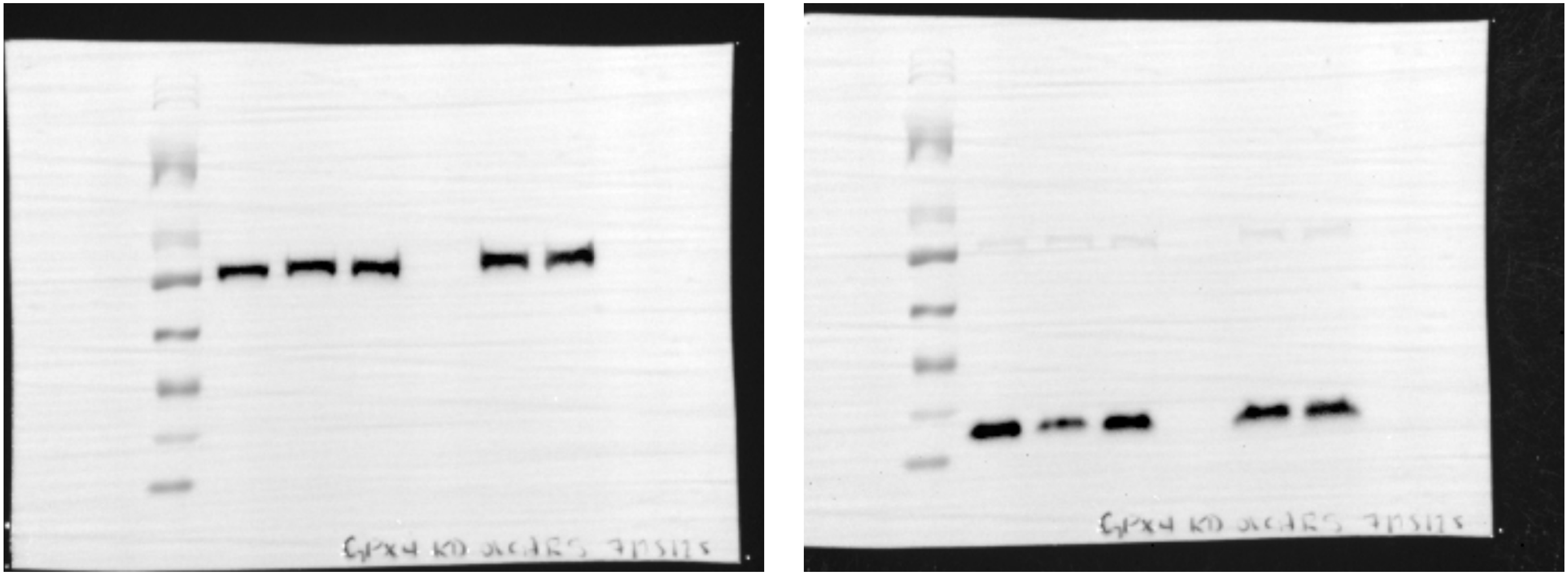

Supplementary Figure 3D

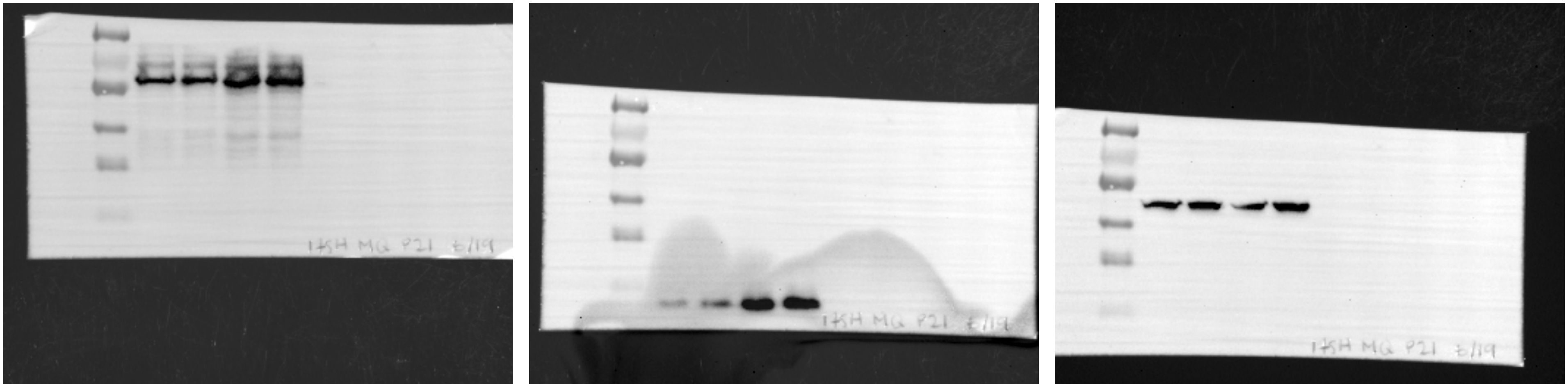

Supplementary Figure 3F

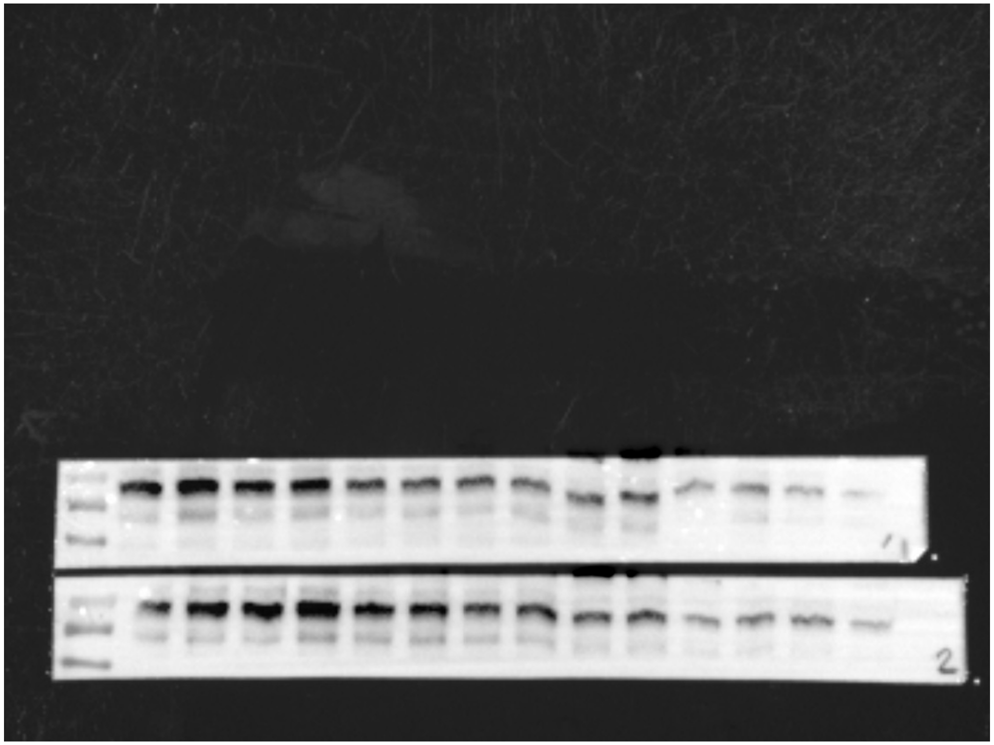

Supplementary Figure 3G

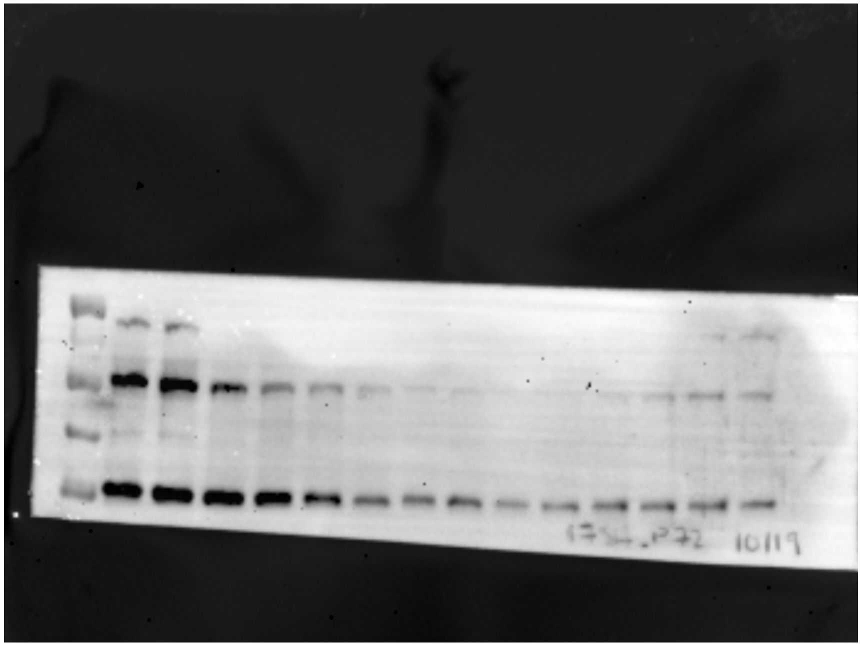

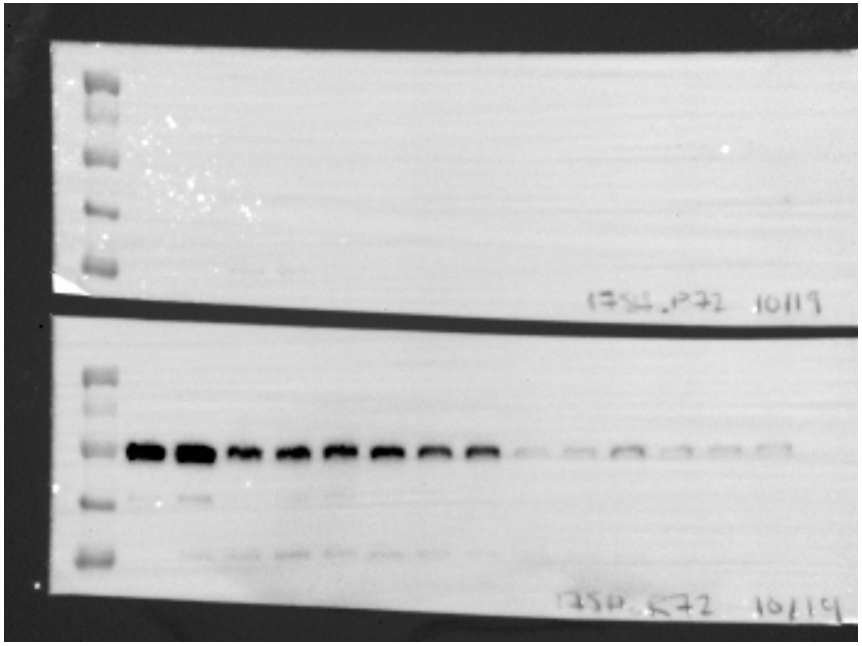

Supplementary Figure 3I

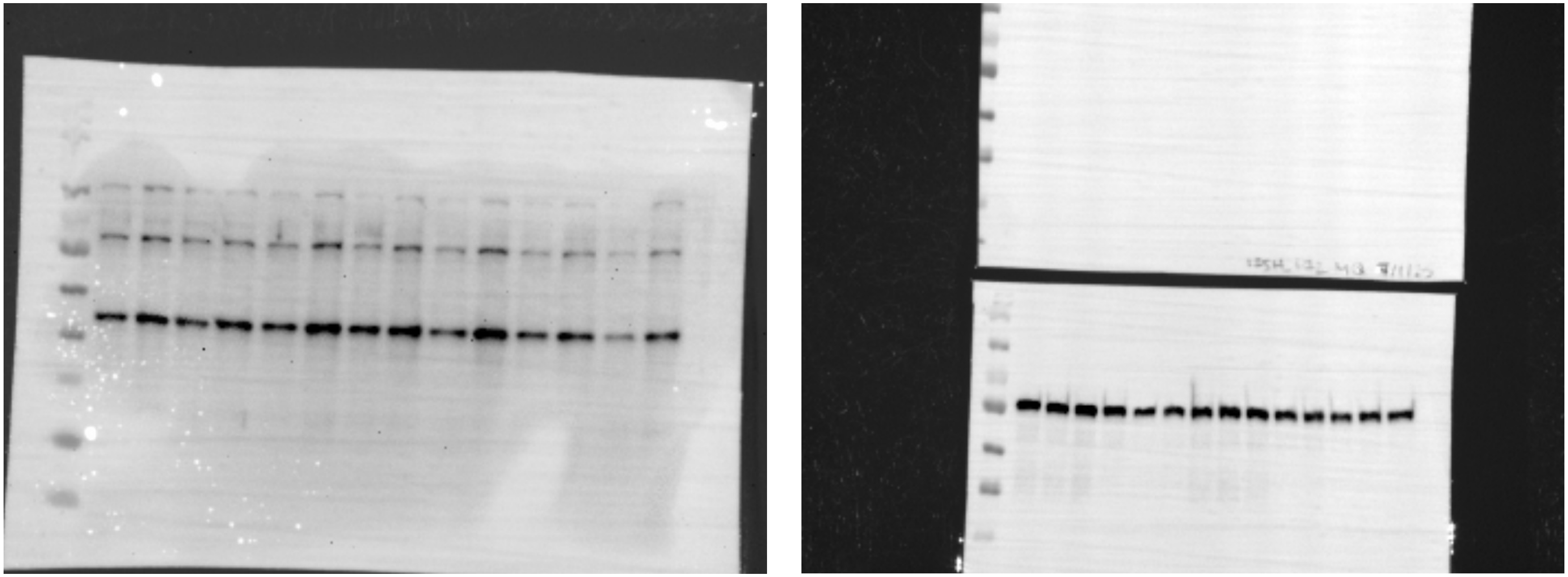

Supplement: Supplementary file 2 — Original Western Blot Images [file 41420_2026_3152_MOESM2_ESM.pdf]
